# Propofol inhibits myocardial injury induced by microvesicles derived from hypoxia-reoxygenated endothelial cells via lncCCT4-2/CCT4 signaling

**DOI:** 10.1186/s40659-023-00428-3

**Published:** 2023-05-05

**Authors:** Xiaojun Zhang, Changsen Wang, Hao Xu, Shuyun Cai, Keyu Liu, Simeng Li, Linming Chen, Siman Shen, Xiaoxia Gu, Jing Tang, Zhengyuan Xia, Zhe Hu, Xiaotang Ma, Liangqing Zhang

**Affiliations:** 1 Department of Anesthesiology, Affiliated Hospital of Guangdong Medical University, Zhanjiang, 524001 China; 2 Key Laboratory of Organ Functional Injury and Protection, Department of Translational Medicine of ZhanJiang, ZhanJiang, 524001 China; 3 Key Laboratory of Autophagy and Major Chronic Non-communicable Diseases of Guangdong, ZhanJiang, 524001 China; 4 Institute of Neurology, Affiliated Hospital of Guangdong Medical University, Zhanjiang, 524001 China; 5 Department of Anaesthesiology, The University of Hong Kong, Pok Fu Lam, Hong Kong SAR, China

**Keywords:** Endothelial microvesicles (EMVs), lncCCT4-2, Ischemia-reperfusion (IR), Cardiomyocyte, Oxidative stress, Apoptosis, Propofol

## Abstract

**Background:**

Ischemia-reperfusion (IR) induces increased release of extracellular vesicles in the heart and exacerbates myocardial IR injury. We have previously shown that propofol attenuates hypoxia/reoxygenation (HR)-induced injury in human umbilical vein endothelial cells (HUVECs) and that microvesicles derived from propofol-treated HUVECs inhibit oxidative stress in endothelial cells. However, the role of microvesicles derived from propofol post-treated HUVECs ((HR + P)-EMVs) in IR-injured cardiomyocytes is unclear. In this study, we aimed to investigate the role of (HR + P)-EMVs in cardiac IR injury compared to microvesicles derived from hypoxic/reoxygenated HUVECs (HR-EMVs) and to elucidate the underlying mechanisms.

**Methods:**

Hypoxia/reoxygenation (HR) models of HUVECs and AC16 cells and a mouse cardiac IR model were established. Microvesicles from HR-injured HUVECs, DMSO post-treated HUVECs and propofol post-treated HUVECs were extracted by ultra-high speed centrifugation, respectively. The above EMVs were co-cultured with HR-injured AC16 cells or injected intracardially into IR mice. Flow cytometry and immunofluorescence were used to determine the levels of oxidative stress and apoptosis in cardiomyocytes. Apoptosis related proteins were detected by Western blot. Echocardiography for cardiac function and Evans blue-TTC staining for myocardial infarct size. Expression of lncCCT4-2 in EMVs and AC16 cells was analysed by whole transcriptome sequencing of EMVs and RT-qPCR. The molecular mechanism of inhibition of myocardial injury by (HR + P)-EMVs was elucidated by lentiviral knockdown of lncCCT4-2, plasmid overexpression or knockdown of CCT4, and actinomycin D assay.

**Results:**

In *vitro* and in *vivo* experiments confirmed that HR-EMVs exacerbated oxidative stress and apoptosis in IR-injured cardiomyocytes, leading to increased infarct size and worsened cardiac function. Notably, (HR + P)-EMVs induced significantly less oxidative stress and apoptosis in IR-injured cardiomyocytes compared to HR-EMVs. Mechanistically, RNA sequencing of EMVs and RT-qPCR showed that lncCCT4-2 was significantly upregulated in (HR + P)-EMVs and cardiomyocytes co-cultured with (HR + P)-EMVs. Reduction of lncCCT4-2 in (HR + P)-EMVs enhanced oxidative stress and apoptosis in IR-injured cardiomyocytes. Furthermore, the anti-apoptotic activity of lncCCT4-2 from (HR + P)-EMVs was achieved by increasing the stability of CCT4 mRNA and promoting the expression of CCT4 protein in cardiomyocytes.

**Conclusions:**

Our study showed that (HR + P)-EMVs uptake by IR-injured cardiomyocytes upregulated lncCCT4-2 in cardiomyocytes and promoted CCT4 expression, thereby inhibiting HR-EMVs induced oxidative stress and apoptosis.

**Supplementary Information:**

The online version contains supplementary material available at 10.1186/s40659-023-00428-3.

## Highlights

Microvesicles from hypoxic/reoxygenated HUVECs (HR-EMVs) exacerbated oxidative stress and apoptosis in IR-injured cardiomyocytes.

Microvesicles from propofol post-treated HUVECs ((HR + P)-EMVs) induced diminished oxidative stress and apoptosis in IR-injured cardiomyocytes compared with microvesicles from hypoxic/reoxygenated HUVECs (HR-EMVs).

lncCCT4-2 was significantly highly expressed in (HR + P)-EMVs and cardiomyocytes co-cultured with (HR + P)-EMVs, and reduction of lncCCT4-2 in (HR + P)-EMVs enhanced oxidative stress and apoptosis in IR-injured cardiomyocytes.

lncCCT4-2 inhibited HR-EMVs induced oxidative stress and apoptosis in HR-injured AC16 cells by increasing the stability of CCT4 mRNA and promoting the expression of CCT4 protein in AC16 cells.

**Supplementary Information:**

The online version contains supplementary material available at 10.1186/s40659-023-00428-3.

## Background

Ischemic heart disease (IHD) is the leading cause of death and disability worldwide, despite current therapeutic advancements [1]. The effects of IHD are typically attributed to the deleterious effects of acute myocardial ischemia-reperfusion injury (IRI) [2]. Various sources produce a burst of oxidative stress in the early stages of myocardial reperfusion. This deleterious oxidative stress mediates myocardial injury and cardiomyocyte death via several mechanisms [3, 4]. Cardiomyocyte death by apoptosis is fundamental pathogenesis of ischemic cardiomyopathy [5, 6]. Endothelial cells are abundant in perfused tissues as “first responders” to hypoxic stress [7]. In the early stages of cardiac reperfusion, IRI first appears in endothelial cells of the small coronary vessels. Reperfusion then induces the release of pro-apoptotic mediators from endothelial cells, which promote apoptosis in IR-injured cardiomyocytes [8].

Endothelial microvesicles (EMVs) are membranous vesicles 0.1-1 μm in diameter that can be released by activated or apoptotic endothelial cells [9]. EMVs can carry ‘cargo’, including RNAs, proteins and lipids, from the parent cells and transfer the cargo to recipient cells, causing phenotypic changes in the recipient cells [9, 10]. The biological composition of EMVs and their biological functions are closely related to the function of endothelial cells in different states [11]. EMVs are effective mediators of communication between the endothelium and the myocardium [11, 12]. It has been shown that EMVs derived from normal endothelial cells can attenuate IRI-induced cardiomyocyte death by supplementing damaged cardiomyocytes with metabolic substances [13]. Conversely, EMVs from endothelial cells exacerbate cardiomyocytes injury via miR-503 during the onset of acute myocardial infarction (AMI) [14]. Similarly, EMVs from HR-treated HUVECs induce apoptosis and oxidative stress in H9C2 cardiomyocytes [15]. Therefore, inhibiting cardiac IR-induced vascular endothelial cell injury, improving endothelial cell function, and blocking harmful microvesicle signalling between endothelial cells and cardiomyocytes may be effective strategies to alleviate cardiac IRI.

Propofol (2, 6-diisopropylphenol) is an intravenous anesthetic agent with antioxidant activity. Propofol has been reported to inhibit ROS-mediated lipid peroxidation and reduce myocardial stress during cardiac surgery [16]. Furthermore, propofol inhibits IRI by reducing oxidative stress, protecting mitochondrial function, and suppressing apoptosis in various experimental models [17–20]. We have previously demonstrated that propofol treatment inhibits HR-induced oxidative stress and autophagy in endothelial cells [21, 22]. We have also previously shown that microvesicles from propofol-pretreated HUVECs inhibit apoptosis of endothelial cells. However, the role of microvesicles derived from propofol post-treated HUVECs ((HR + P)-EMVs) in IR-injured cardiomyocytes is unclear.

There is growing evidence that lncRNAs in microvesicles play an important role in cardiovascular disease (CVD) [23]. For instance, lncRNAs are involved in the early development of atherosclerosis [24], the occurrence of AMI [25] and IRI [26]. In this study, RNA sequencing of EMVs revealed a significant increase in lncCCT4-2 expression in microvesicles (HR + P)-EMVs derived from HUVECs post-treated with propofol. Antisense lncRNAs often regulate the expression of their overlapping sense protein-coding genes in cis through various transcription-dependent mechanisms [27, 28]. As a target gene of lncCCT4-2, chaperonin containing t-complex 4 (CCT4) is the antisense gene of lncCCT4-2. CCT (Chaperonin Containing TCP1) is the obligate chaperone for many essential proteins, which is capable of folding a subset of essential and topologically complex proteins, including cell cycle regulators, signaling proteins, and cytoskeletal components [29, 30]. CCT4 is a subunit of CCT, and knockdown of CCT4 has been shown to induce apoptosis of tumor cells [31, 32], impaired organ development, and even lethality in Drosophila [33]. However, the effect of CCT4 on cardiomyocytes is unclear. Here, we investigated the effects of HR-EMVs and (HR + P)-EMVs on IR-injured hearts and explored the underlying mechanisms through lncCCT4-2/CCT4 signaling.

## Results

### Characterization and internalization of endothelial microvesicles

**Fig. 1:**
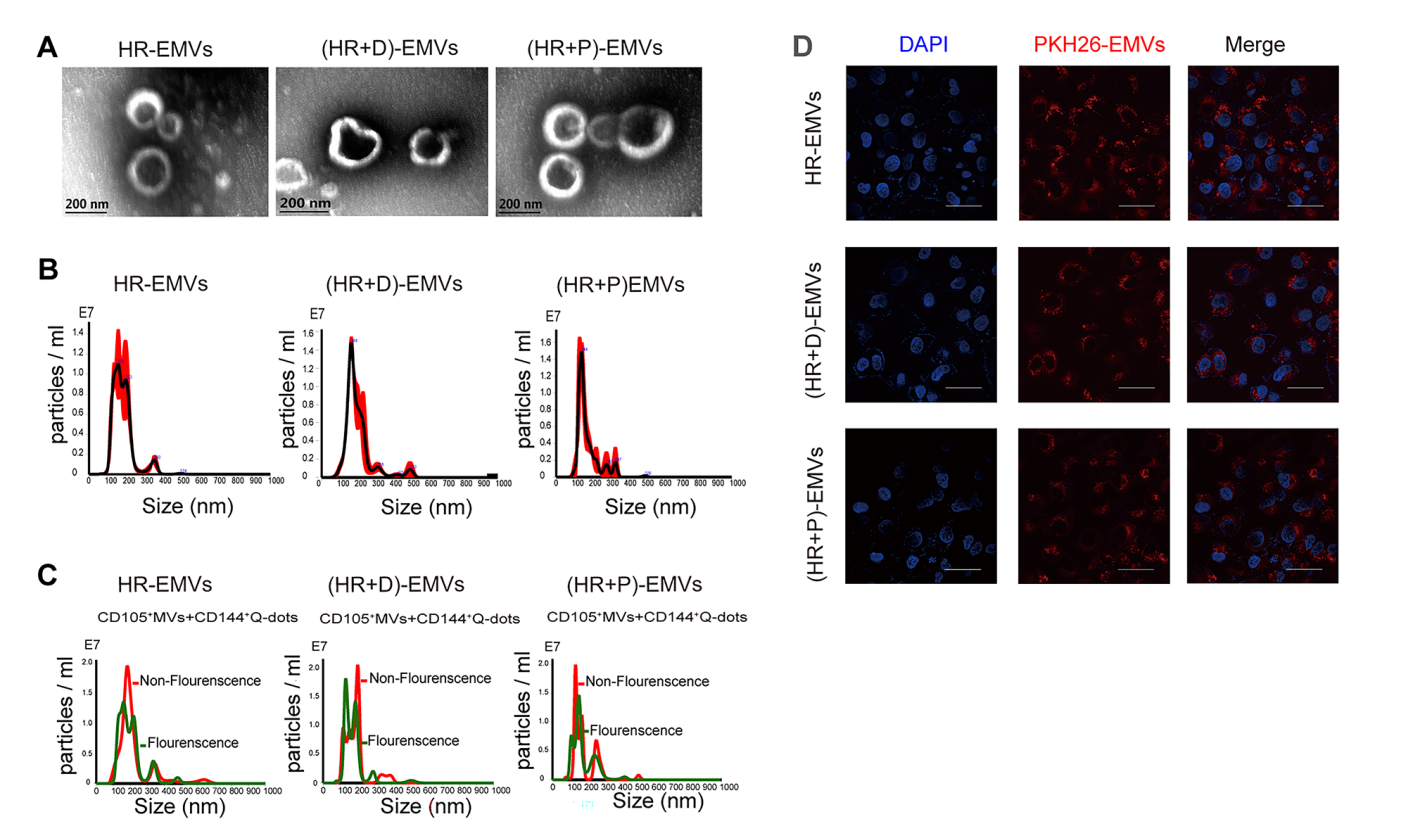
Characterization and internalization of endothelial microvesicles. **(A)** Representative TEM image of HR-EMVs, (HR + D)-EMVs and (HR + P)-EMVs. Scale bars, 200 nm. TEM, transmission electron microscopy. **(B-C)** Nanoparticle tracking analysis (NTA) plots show the size / concentration distribution of HR-EMVs, (HR + D)-EMVs and (HR + P)-EMVs. CD105^+^ +CD144^+^ Q-dots EMVs under fluorescence / nonfluorescence modes. Red curve, CD105^+^ +CD144^+^ Q-dots EMVs were measured under light scatter (nonfluorescence) mode. Green curve, CD105^+^ +CD144^+^ Q-dots EMVs were measured under fluorescence mode. CD105, specific for endothelial cells. CD144, vascular endothelial antigen. **(D)** Immunofluorescent staining showing the uptake of EMVs by AC16 cells after coculture with PKH26-labelled EMVs for 24 h. Scale bars, 100 μm. HR, hypoxia/reoxygenation; EMVs, endothelial microvesicles

Microvesicles from hypoxic/reoxygenated HUVECs (HR-EMVs), microvesicles from DMSO post-treated HUVECs (HR + D)-EMVs and microvesicles from propofol post-treated HUVECs (HR + P)-EMVs were extracted, respectively. Transmission electron microscopy (TEM) revealed isolated pellets with typical spherical and bilayer-membrane structures (Fig. 1A). Nanoparticle tracking analysis (NTA) results showed that the size of EMVs from the three groups all ranged from 100 to 700 nm in diameter, which is within the size range of microvesicles (Fig. 1B). There was no significant difference in the morphology and size of EMVs among the three groups. To identify these microvesicles derived from endothelial cells, we incubated bead-captured microvesicles with endothelial cell markers (CD144 and CD105), and the fluorescence NTA results revealed that the detection rate of CD105^+^ +CD144^+^ EMVs was approximately 76% (Fig. 1C). PKH26 labeled EMVs were cultured with AC16 cells for 24 h and photographed, the fluorescence images revealed that AC16 cells displayed red fluorescence (Fig. 1D), indicating that the EMVs were taken up by AC16 cells.

### (HR + P)-EMVs showed less effects on inducing oxidative stress and apoptosis compared with HR-EMVs in hypoxia-reoxygenated cardiomyocytes

**Fig. 2:**
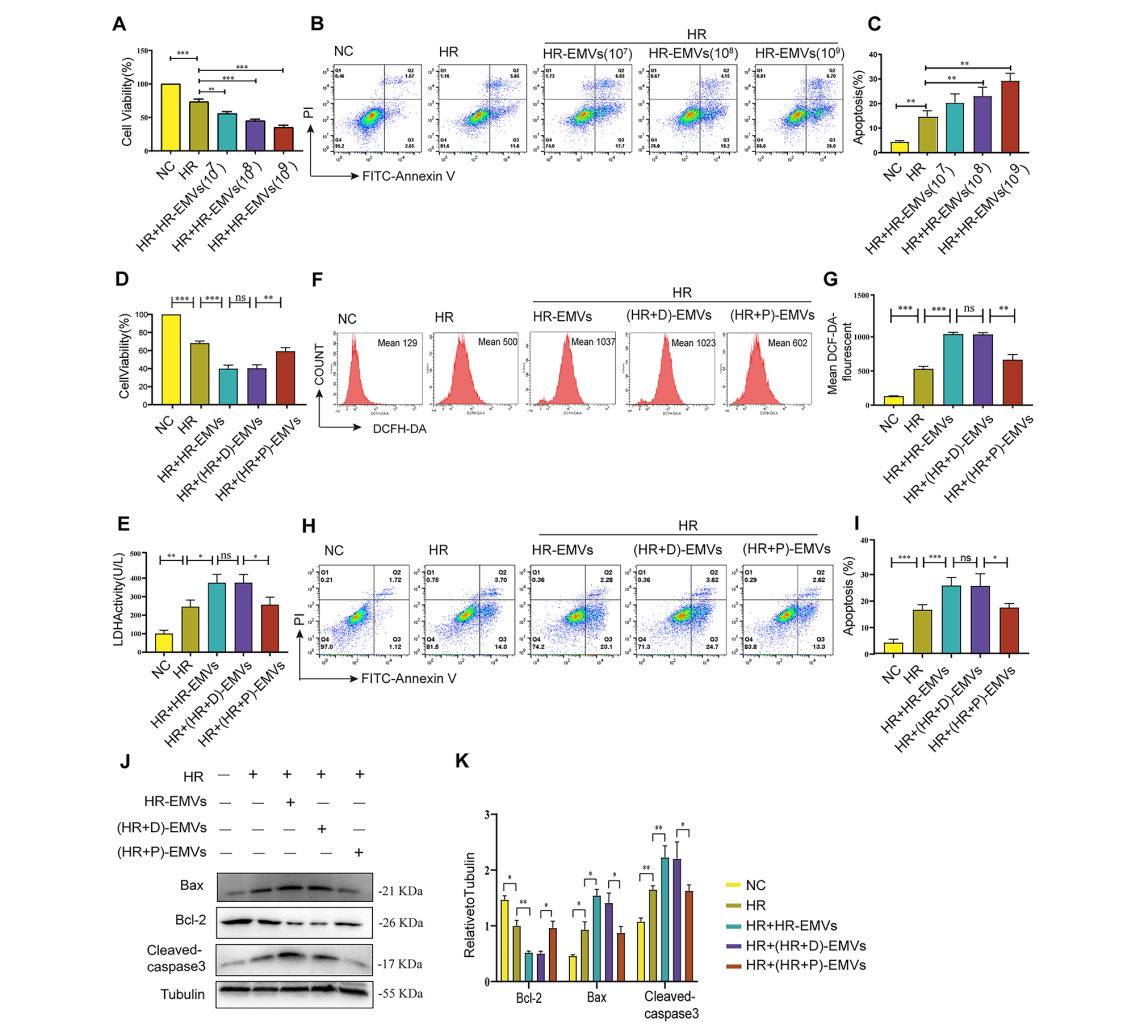
(HR + P)-EMVs showed less effects on inducing oxidative stress and apoptosis compared with HR-EMVs in hypoxia-reoxygenated cardiomyocytes. HR-injured AC16 cells were co-cultured with different concentrations (10^7, 10^8 and 10^9 particles/ml) of HR-EMVs for 24 h: **(A)** CCK-8 assay for AC16 cell viability. **(B)** Flow cytometric detection of AC16 cell apoptosis by Annexin V-FITC/PI double staining. **(C)** Quantitative analysis of apoptotic cells. Cells of annexin V+/PI − or annexin V+/PI + were added together to calculate the percentage of apoptotic cells. HR-injured AC16 cells were co-cultured with 10^9 particles/ml of HR-EMVs, (HR + D)-EMVs and (HR + P)-EMVs for 24 h: **(D)** CCK-8 assay for AC16 cell viability. **(E)** LDH assay for lactate dehydrogenase activity in the cell supernatant. **(F)** Flow cytometry detection of reactive oxygen species (ROS) levels in AC16 cells after DCFH-DA staining. **(G)** Quantitative analysis of ROS. **(H)** Flow cytometry detection of apoptotic rate in AC16 cells after Annexin V-FITC/PI double staining. **(I)** Quantitative analysis of apoptotic cells. **(J-K)** Western blotting analysis of Bax, Bcl-2, cleaved caspase-3 in AC16 cells, and the relative gray scales calculated by ImageJ software. These data were representative results (n = 3) of three repetitions. *P < 0.05, **P < 0.01, ***P < 0.001, ns, not significant. Data are expressed as mean ± SEM.

To confirm that HR-EMVs induced cardiomyocyte injury, normoxic and hypoxic AC16 cells were co-cultured with different concentrations (10^7, 10^8, and 10^9 particles/mL) of HR-EMVs for 24 h. The viability and apoptosis of normoxic and hypoxic AC16 cells were examined by CCK-8 assay and Annexin V-FITC/PI staining. Compared with the HR group, the viability of HR-injured AC16 cells treated with HR-EMVs was significantly decreased (Fig. 2A) and the apoptosis rate was significantly increased (Fig. 2B and C), and HR-EMVs at 10^9 particles/ml showed the most significant damaging effect on cardiomyocytes. Therefore, EMVs co-cultured with AC16 cells in subsequent experiments at a concentration of 10^9 particles/mL. Additional experimental data showed that LDH activity (Fig. 2E), ROS levels (Fig. 2F and G) and the protein levels of pro-apoptotic proteins (Bax/Bcl-2 and cleaved caspase-3) (Fig. 2J and K) were significantly increased in AC16 cells in the HR-EMVs treated group compared with the HR group. However, under normoxic conditions, HR-EMVs had no significant damaging effect on AC16 cells. There was no significant difference in cell viability (Fig. S1A) and apoptosis rate (Figs. S1B-C) in the HR-EMVs treated group compared with the normal control (NC) group. These results confirmed that HR-EMVs had no damaging effect on normal AC16 cells, but significantly exacerbated oxidative stress and apoptosis in HR-injured AC16 cells.

Hypoxic/reoxygenated HUVECs were treated with or without propofol at concentrations of 25 μm, 50 μm, 100 μm, and 150 μm to obtain HR-EMVs, (HR + P25)-EMVs, (HR + P50)-EMVs, (HR + P100)-EMVs, and (HR + P150)-EMVs, and then hypoxic/reoxygenated AC16 cells were co-cultured with the above EMVs for 24 h. Compared with the HR-EMVs treatment group, (HR + P50)-EMVs and (HR + P100)-EMVs significantly increased cell viability (Fig. S1D). In addition, we further examined the anti-apoptotic ability of (HR + P50)-EMVs and (HR + P100)-EMVs by flow cytometry, and both EMVs significantly reduced the apoptosis rate of AC16 cells compared with the HR-EMVs treated group, while the anti-apoptotic effect of (HR + P100)-EMVs was more significant (Figs. S1E-F). Therefore, we further investigated the function of EMVs derived from 100 μm propofol post-treated HUVECs. DMSO was the solvent of propofol, and DMSO post-treatment of HUVECs derived EMVs ((HR + D)-EMVs) was used as the control group to exclude the effect of DMSO.

Compared with the HR-EMVs and (HR + D)-EMVs treated groups, CCK-8 and LDH assays showed that (HR + P)-EMVs significantly attenuated the damaging effects onfurther investigated AC16 cells (Fig. 2D and E), and flow cytometry showed that (HR + P)-EMVs significantly inhibited cardiomyocyte ROS levels (Fig. 2F and G) and reduced the apoptotic rate (Fig. 2H and I). Moreover, the pro-apoptotic protein expression of Bax and cleaved caspase-3 was significantly inhibited, while that of Bcl-2 was significantly increased in the (HR + P)-EMVs treated group compared with HR-EMVs and (HR + D)-EMVs treated groups (Fig. 2J and K). These results suggest that the oxidative stress and apoptotic effects of (HR + P)-EMVs on hypoxia-reoxygenated cardiomyocytes are significantly attenuated compared with HR-EMVs.

### (HR + P)-EMVs showed less effects on inducing cardiac injury compared with HR-EMVs in ischemia-reperfused hearts

**Fig. 3:**
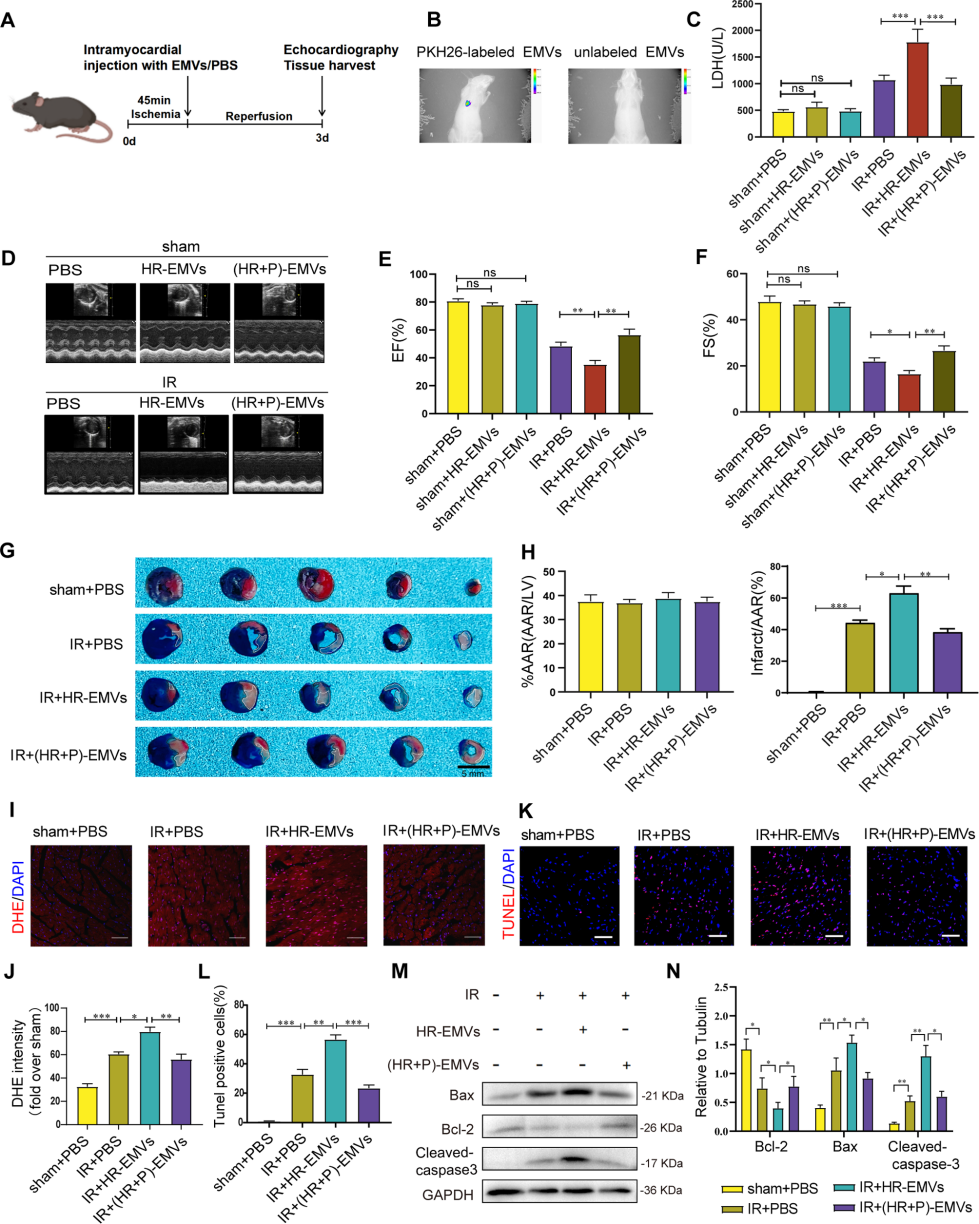
(HR + P)-EMVs showed less effects on inducing cardiac injury compared with HR-EMVs in ischemia-reperfused hearts. **(A)** Protocol schematic for infusion of EMVs and tissue harvest. **(B)** Bioluminescence imaging showing uptake of PKH26-labelled EMVs by cardiomyocytes. Mice were randomly divided into 6 groups: sham/IR + PBS, sham/IR + HR-EMVs, sham/IR+(HR + P)-EMVs, 5 mice per group, and 50 ul EMVs (10^9 particles/ml) were injected intracardially: **(C)** Plasma lactate dehydrogenase (LDH) activity in mice. **(D)** Echocardiographic assessment of cardiac function. **(E)** Quantitative analysis of left ventricular ejection fraction (LVEF). **(F)** Quantitative analysis of left ventricular shortening fraction (LVFS). Mice were randomly divided into 4 groups: sham + PBS, IR + PBS, IR + HR-EMVs, IR + (HR + P)-EMVs, 5 mice per group,and 50 ul EMVs (10^9 particles/ml) were injected intracardially: **(G)** Evans blue-TTC staining for detection of myocardial infarct area. Area-at-risk (AAR; black line), infarct size (white dotted line), scale bar = 5 mm. **(H)** Quantitative analysis of the percentage AAR and percentage infarct of hearts in (G). **(I)** Myocardial tissue ROS levels were detected by fluorescence microscopy after DHE staining of heart sections. **(J)** Quantitative analysis of DHE staining. **(K)** Fluorescence microscopy of cardiac sections after TUNEL staining to detect apoptosis levels in cardiac myocytes. **(L)** Quantification of TUNEL staining. **(M-N)** Western blotting analysis was performed for Bax, Bcl-2, and cleaved caspase-3 in heart tissue, and relative grayscale was calculated by ImageJ software. These data were representative results (n = 5) of five repetitions. *P < 0.05, **P < 0.01, ***P < 0.001, ns, not significant. Data are expressed as mean ± SEM.

To further clarify the role of HR-EMVs and (HR + P)-EMVs in cardiac IR injury, HR-EMVs, (HR + P)-EMVs and equal volumes of PBS were transfused into sham or IR-injured heart (Fig. 3A). Bioluminescence imaging showed that PKH26-labeled EMVs injected into the myocardium could be internalized by myocardial tissue uptake. (Fig. 3B). We demonstrated in vivo that HR-EMVs exacerbated IR-induced cardiac injury.

Plasma LDH was significantly increased (Fig. 3C) and LVEF and LVFS were decreased (Fig. 3D-F) in the IR + HR-EMVs group compared with the IR + PBS mice. However, there were no significant differences in plasma LDH (Fig. 3C) and cardiac function (LVEF and LVFS) (Fig. 3D-F) between the sham + PBS and sham + HR-EMVs groups. Myocardial infarction size (expressed as percentage of area at risk, Fig. 3G-H), ROS levels (Fig. 3I and J), and TUNEL-positive cell rate (Fig. 3K and L) were significantly increased in the IR + HR-EMVs mice compared with the IR + PBS group, with a concomitant increase in the expression of the pro-apoptotic proteins Bax and cleaved caspase-3 and a significant decrease in Bcl2 (Fig. 3M and N). These results indicate that HR-EMVs increase IR-induced oxidative stress and apoptosis in cardiomyocytes and exacerbate cardiac injury.

Noteworthy, (HR + P)-EMVs attenuated the oxidative stress and apoptotic effects mediated by HR-EMVs in cardiomyocytes. Compared with the IR + HR-EMVs group, the IR+(HR + P)-EMVs group had reduced plasma LDH levels (Fig. 3C), significantly increased cardiac LVEF and LVFS (Fig. 3D-F), with concomitant significant reduction in myocardial infarct size (Fig. 3G-H). Compared with the HR-EMVs injection group, (HR + P)-EMVs infusion inhibited ROS levels (Fig. 3I and J) and TUNEL-positive cell rates (Fig. 3K and L) in cardiomyocytes. In addition, IR+(HR + P)-EMVs decreased Bax and cleaved caspase-3 protein levels and increased Bcl2 expression in mice compared with the IR + HR-EMVs group (Fig. 3M and N). Overall, we verified in vivo that (HR + P)-EMVs showed less effects on inducing cardiac injury compared with HR-EMVs in ischemia-reperfused hearts.

### LncCCT4-2 in (HR + P)-EMVs is a potent mediator to inhibit oxidative stress and apoptosis in cardiomyocytes

**Fig. 4:**
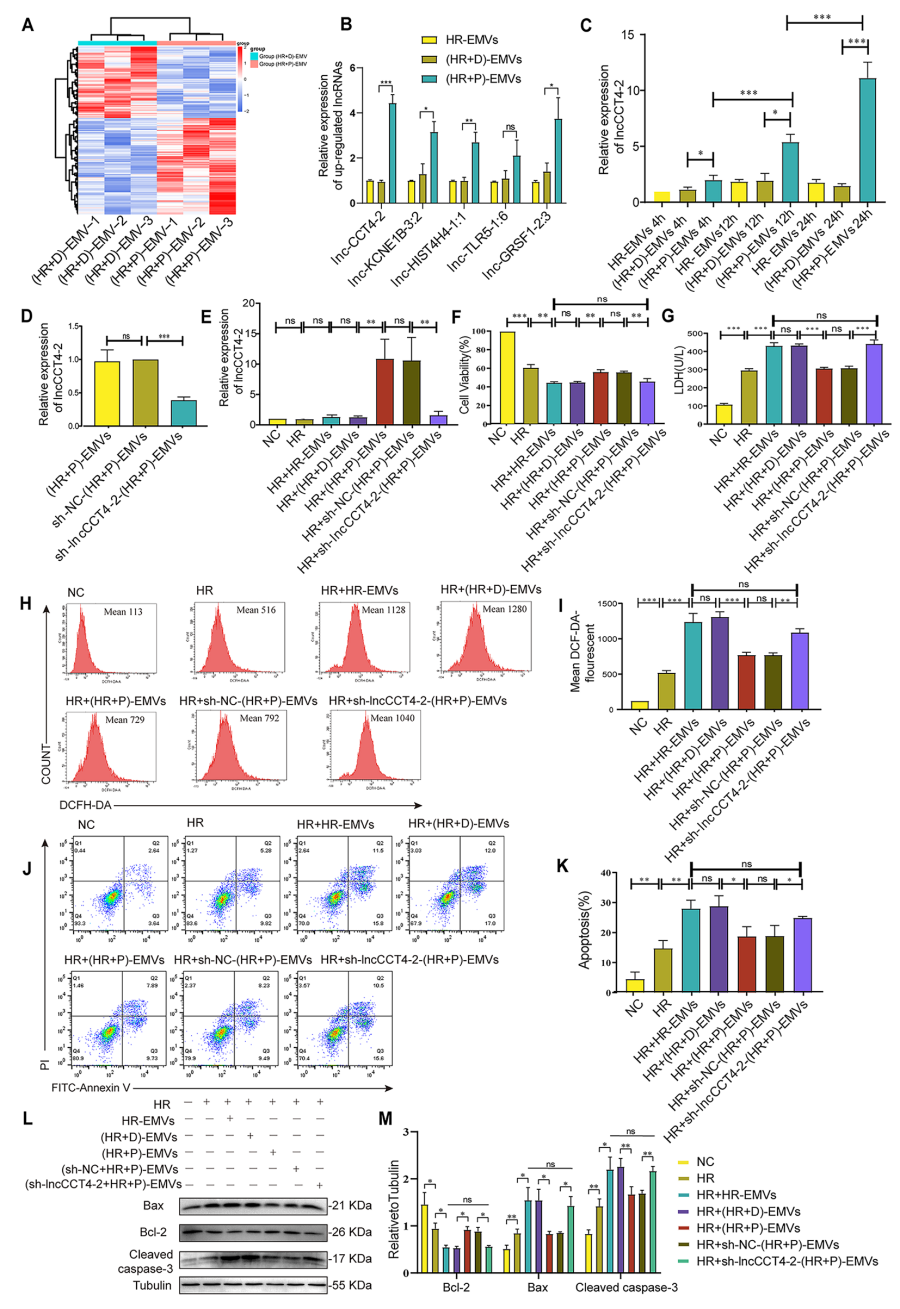
LncCCT4-2 in (HR + P)-EMVs is a potent mediator to inhibit oxidative stress and apoptosis in cardiomyocytes. **(A)** Heat map of differentially expressed microvesicular lncRNAs in (HR + D)-EMVs and (HR + P)-EMVs. **(B)** Analysis of differentially expressed lncRNAs in HR-EMVs, (HR + D)-EMVs and (HR + P)-EMVs by RT-qPCR. **(C)** HR-injured AC16 cells were co-cultured with HR-EMVs, (HR + D)-EMVs and (HR + P)-EMVs for 4 h, 12 and 24 h, respectively, and RT-qPCR was performed to detect expression of lncCCT4-2 in AC16 cells. **(D)** RT-qPCR analysis of lncCCT4-2 knockdown efficiency in sh-lncCCT4-2-(HR + P)-EMVs. HR-injured AC16 cells were co-cultured with HR-EMVs, (HR + D)-EMVs, (HR + P)-EMVs and sh-lncCCT4-2-(HR + P)-EMVs for 24 h, respectively: **(E)** Expression of lncCCT4-2 in AC16 cells was analyzed by RT-qPCR. **(F)** CCK-8 assay for AC16 cell activity. **(G)** Lactate dehydrogenase (LDH) activity of AC16 cell supernatants. **(H)** Flow cytometry detection of reactive oxygen species (ROS) levels in AC16 cells after DCFH-DA staining. **(I)** Quantitative analysis of ROS. **(J)** Flow cytometry detection of apoptotic rate in AC16 cells after Annexin V-FITC/PI double staining. **(K)** Quantitative analysis of apoptotic cells. **(L-M)** Western blotting analysis of Bax, Bcl-2, cleaved caspase-3 in AC16 cells, and the relative gray scales calculated by ImageJ software. These data were representative results (n = 3) of three repetitions. *P < 0.05, **P < 0.01, ***P < 0.001, ns, not significant. Data are expressed as mean ± SEM.

To explore the mechanism by which (HR + P)-EMVs alleviate myocardial injury, lncRNAs in EMVs were analyzed by RNA sequencing. The expression of lncRNAs in (HR + D)-EMVs and (HR + P)-EMVs was significantly different, with 96 lncRNAs significantly up-regulated and 71 lncRNAs significantly down-regulated (Fig. 4A). Among the significantly up-regulated lncRNAs, we selected five lncRNAs with high expression abundance in (HR + P)-EMVs for qPCR validation, and found that lncCCT4-2 was most significantly up-regulated in (HR + P)-EMVs with the most stable expression (Fig. 4B). The NONCODE (http://www.noncode.org/) and LNcipedia (https://lncipedia.org/) databases show that lncCCT4-2 is an antisense lncRNA, 2328 base pairs (bp) long, which is located on chromosome 2 (Additional file 3). Meanwhile, the NONCODE database showed that lncCCT4-2 is highly expressed in extracellular vesicles secreted by HUVECs, but low in human heart. To confirm that (HR + P)-EMVs increased the expression of lncCCT4-2 in cardiomyocytes, AC16 cells were co-cultured with HR-EMVs, (HR + D)-EMVs and (HR + P)-EMVs for 4 h, 12 and 24 h, respectively, and then the expression of lncCCT4-2 in AC16 cells was detected by qPCR. Notably, lncCCT4-2 was upregulated in AC16 cells cultured with (HR + P)-EMVs compared with HR-EMVs and (HR + D)-EMVs treatment groups (Fig. 4C). Furthermore, the expression of lncCCT4-2 in AC16 cells was positively correlated with the co-culture time of (HR + P)-EMV, and the most significant increase in lncCCT4-2 was observed at 24 h of co-culture (Fig. 4C).

HUVECs were infected with lentivirus containing sh-lncCCT4-2 to establish HUVECs cell lines with stable knockdown of lncCCT4-2, followed by propofol post-treatment to obtain EMVs with reduced lncCCT4-2 expression, i.e. sh-lncCCT4-2-(HR + P)-EMVs. Expression of lncCCT4-2 in sh-lncCCT4-2-(HR + P)-EMVs and co-cultured AC16 cells was examined by qPCR. The level of lncCCT4-2 was significantly lower in sh-lncCCT4-2-(HR + P)-EMVs compared with sh-NC-(HR + P)-EMVs (Fig. 4D). The expression of lncCCT4-2 in AC16 cells co-cultured with sh-lncCCT4-2-(HR + P)-EMVs was also correspondingly reduced compared with sh-NC-(HR + P)-EMVs treated group (Fig. 4E).

HR-injured AC16 cells were co-cultured with HR-EMVs, (HR + D)-EMVs, (HR + P)-EMVs, sh-NC-(HR + P)-EMVs and sh-lncCCT4-2-(HR + P)-EMVs for 24 h, respectively. Compared with the (HR + P)-EMVs treated group, we noticed a decrease in cell viability (Fig. 4F), an increase in LDH activity (Fig. 4G), and an elevation in ROS levels (Fig. 4H and I) and apoptosis rate (Fig. 4J and K) in the sh-lncCCT4-2-(HR + P)-EMVs treated group of AC16 cells. In addition, the pro-apoptosis protein expression of Bax and cleaved caspase-3 were elevated, while anti-apoptosis Bcl-2 was declined in the sh-lncCCT4-2-(HR + P)-EMVs-treated group compared with the (HR + P)-EMVs-treated group (Fig. 4L and M). These results collectively indicate that (HR + P)-EMVs silencing lncCCT4-2 exhibit similar cardiomyocyte damaging effects to HR-EMVs. Therefore, lncCCT4-2 may be an important mediator of inhibition of oxidative stress and apoptosis in cardiomyocytes.

### Knockdown of CCT4 caused (HR + P)-EMVs to show enhanced oxidative stress and apoptosis in hypoxia-reoxygenated cardiomyocytes

**Fig. 5:**
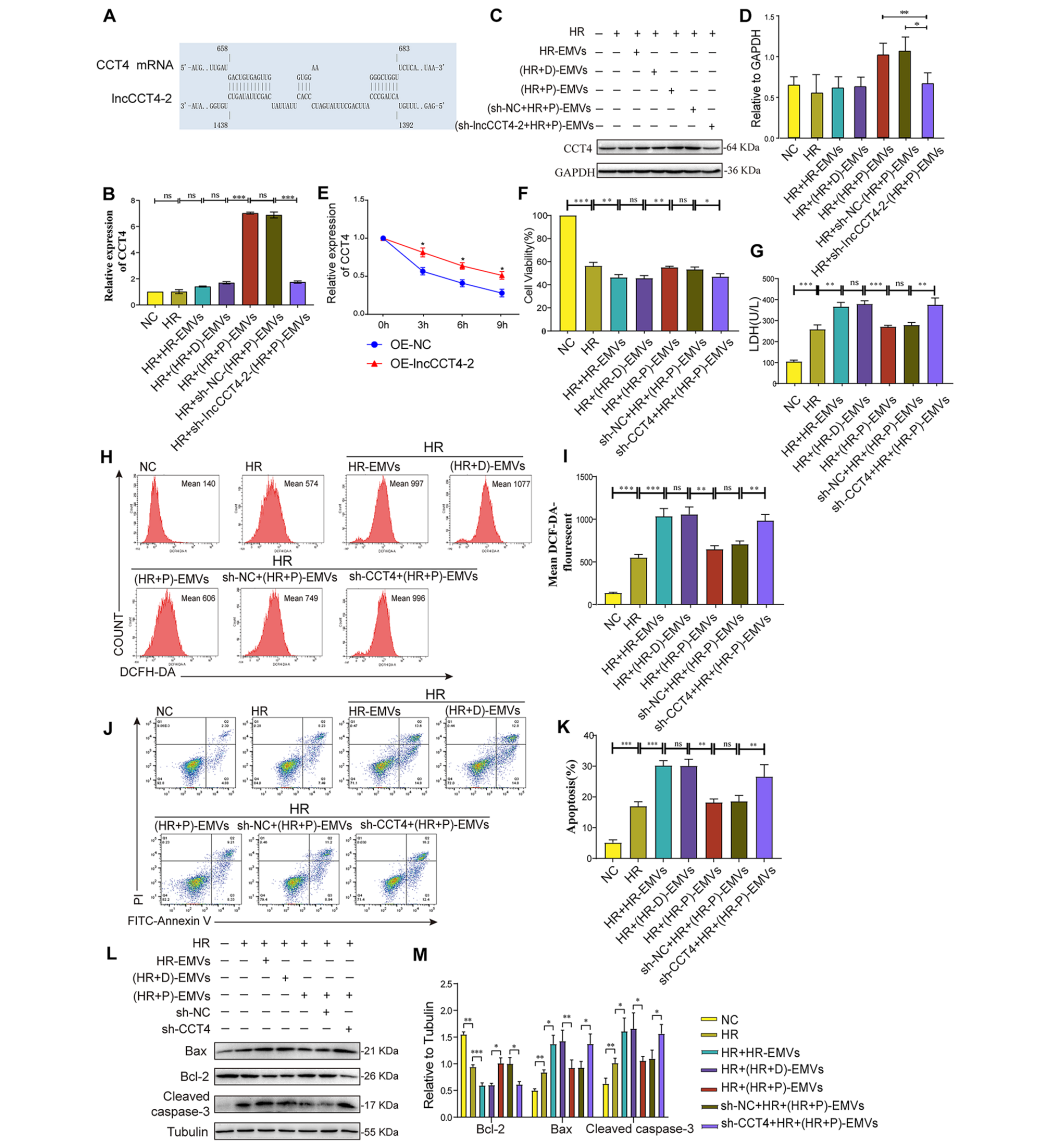
Knockdown of CCT4 caused (HR + P)-EMVs to show enhanced oxidative stress and apoptosis in hypoxia-reoxygenated cardiomyocytes. **(A)** IntaRNA predicted base sites of lncCCT4-2 binding to CCT4. HR-injured AC16 cells were co-cultured with HR-EMVs, (HR + D)-EMVs, (HR + P)-EMVs and sh-lncCCT4-2-(HR + P)-EMVs, respectively, for 24 h: **(B)** RT-qPCR analysis for CCT4 mRNA expression in AC16 cells. **(C)** Western blotting analysis of CCT4 protein expression in AC16 cells. **(D)** Quantitative analysis of CCT4 protein expression. **(E)** Overexpression of lncCCT4-2 in AC16 cells, actinomycin D and RT-qPCR to detect the stability of CCT4 mRNA. Knockdown of CCT4 expression in AC16 cells and detection of the effect of (HR + P)-EMVs on HR-injured AC16 cells: **(F)** CCK-8 assay for AC16 cell viability; **(G)** LDH assay for cell supernatant lactate dehydrogenase activity; **(H)** Flow cytometry detection of reactive oxygen species (ROS) levels in AC16 cells after DCFH-DA staining. **(I)** Quantitative analysis of ROS. **(J)** Flow cytometry detection of apoptotic rate in AC16 cells after Annexin V-FITC/PI double staining. **(K)** Quantitative analysis of apoptotic cells. **(L-M)** Western blotting analysis of Bax, Bcl-2, cleaved caspase-3 in AC16 cells, and the relative gray scales calculated by ImageJ software. These data were representative results (n = 3) of three repetitions. *P < 0.05, **P < 0.01, ***P < 0.001, ns, not significant. Data are expressed as mean ± SEM.

CCT4 is a potential cis-regulatory coding gene for lncCCT4-2, and the RNA interactions analysis tool IntaRNA showed that lncCCT4-2 can bind to CCT4 target (Fig. 5A). To explore the relationship between (HR + P)-EMVs-derived lncCCT4-2 and CCT4 regulation, AC16 cells were co-cultured with five different groups of EMVs for 24 h, and then the expression of CCT4 mRNA and CCT4 protein in AC16 cells were detected by RT-qPCR and Western blot, respectively. The results showed that both CCT4 mRNA and CCT4 protein were significantly up-regulated in the (HR + P)-EMVs treated group compared with the HR-EMVs and (HR + D)-EMVs-treated groups (Fig. 5B-D). However, in AC16 cells co-cultured with sh-lncCCT4-2-(HR + P)-EMVs, CCT4 mRNA and CCT4 protein were significantly down-regulated compared to the (HR + P)-EMVs treatment group (Fig. 5B-D).

The expression of CCT4 in cardiomyocytes was consistent with the expression pattern of lncCCT4-2. To elucidate the regulatory mechanism, AC16 cells overexpressing lncCCT4-2 were treated with actinomycin D to suppress transcription, and the loss of CCT4 mRNA expression was measured. LncCCT4-2 prolonged the half-life of CCT4 mRNA (Fig. 5E), suggesting that lncCCT4-2 is required for the regulation of CCT4 mRNA stability. These results show that lncCCT4-2 increased the stability of CCT4 mRNA and the expression of CCT4 protein.

CCT4 has been reported to regulate tumor cell growth and apoptosis as well as organ development [31–33]. However, its function in cardiomyocytes remains unclear. CCT4 knockdown AC16 cells were co-cultured with each group of EMVs for 24 h. The results showed a significant decrease in cell viability (Fig. 5F), an obvious increase in LDH activity (Fig. 5G), and a marked increase in ROS levels (Fig. 5H and I) and apoptosis rate (Fig. 5J and K) in the sh-CCT4 + HR+(HR + P)-EMVs group compared with the sh-NC + HR+(HR + P)-EMVs group. Western blotting also showed the same experimental results, with pro-apoptotic proteins Bax and cleaved caspase-3 expression increased, whereas the anti-apoptotic Bcl-2 was decreased (Fig. 5L and M) in the sh-CCT4 + HR+(HR + P)-EMVs group compared with the sh-NC + HR+(HR + P)-EMVs group. These results suggest that the inhibition of oxidative stress and apoptosis in cardiomyocytes by (HR + P)-EMVs is reduced after CCT4 knockdown. 

### Overexpression of CCT4 abolishes the exacerbating effect of (HR + P)-EMVs silencing lncCCT4-2 on cardiomyocyte injury

**Fig. 6:**
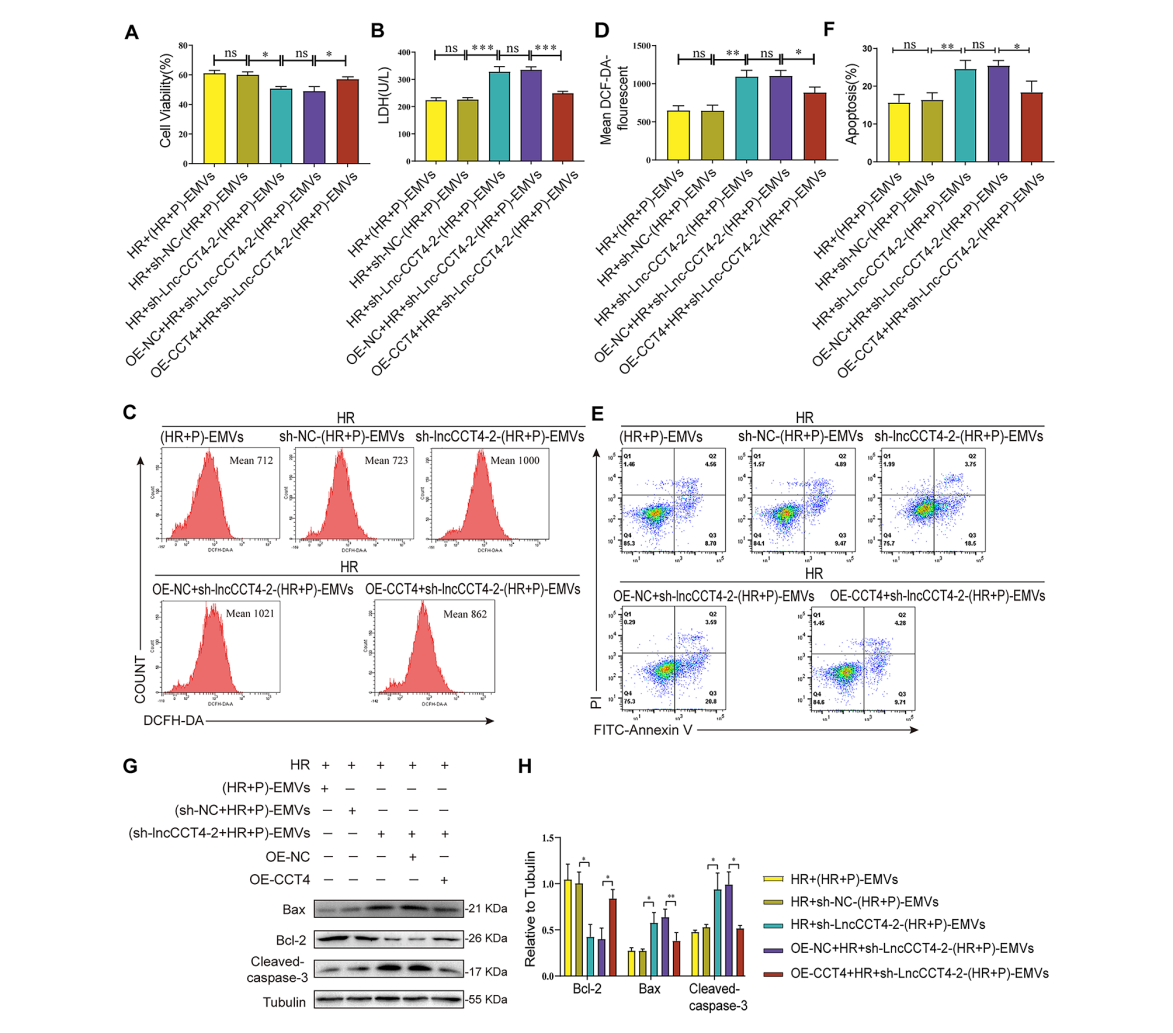
Overexpression of CCT4 abolishes the exacerbating effect of (HR + P)-EMVs silencing lncCCT4-2 on cardiomyocyte injury. Overexpression of CCT4 in AC16 cells and detection of the effect of sh-lncCCT4-2-(HR + P)-EMVs on HR-injured AC16 cells: **(A)** CCK-8 assay for AC16 cell viability. **(B)** LDH assay for AC16 cell supernatant lactate dehydrogenase activity. **(C)** Flow cytometry detection of reactive oxygen species (ROS) levels in AC16 cells after DCFH-DA staining. **(D)** Quantitative analysis of ROS. **(E)** Flow cytometry detection of apoptotic rate in AC16 cells after Annexin V-FITC/PI double staining. **(F)** Quantitative analysis of apoptotic cells. **(G-H)** Western blotting analysis of Bax, Bcl-2, cleaved caspase-3 in AC16 cells, and the relative gray scales calculated by ImageJ software. These data were representative results (n = 3) of three repetitions. *P < 0.05, **P < 0.01, ***P < 0.001, ns, not significant. Data are expressed as mean ± SEM.

To further confirm that lncCCT4-2 derived from (HR + P)-EMVs inhibits cardiomyocyte apoptosis by regulating CCT4 expression. AC16 cells overexpressing CCT4 were co-cultured with each group of EMVs for 24 h. The results showed a significant increase in cell activity (Fig. 6A), an obvious decrease in LDH activity (Fig. 6B), and a significant decrease in ROS levels (Fig. 6C and D) and apoptosis rates (Fig. 6E and F) in the OE-CCT4 + HR+(sh-lncCCT4-2 + HR + P)-EMVs group compared with the OE-NC + HR+(sh-lncCCT4-2 + HR + P)-EMVs group. Western blotting also showed the same results, with decreased expression of pro-apoptotic protein Bax and cleaved caspase-3 and increased expression of the anti-apoptotic protein Bcl-2 in the OE-CCT4 + HR+(sh-lncCCT4-2 + HR + P)-EMVs group compared with the OE-NC + NC+(sh-lncCCT4-2 + HR + P)-EMVs group (Fig. 6G and H). These results suggest that (HR + P)-EMVs derived lncCCT4-2 inhibits oxidative stress and apoptosis in cardiomyocytes by upregulating CCT4 expression in cardiomyocytes.

## Discussion

Cardiovascular disease (CVD) is a major public health problem and social burden worldwide, with a progressively younger age of onset [34]. The reperfusion after cardiac ischemia can lead to further myocardial damage, known as myocardial ischemia/reperfusion injury (MIRI) [35]. Although percutaneous coronary intervention (PCI) and coronary artery bypass grafting have become the most effective methods to salvage surviving myocardium, MIRI caused by the treatment will further exacerbate ischemic myocardial cell injury and reduce the benefit of reperfusion therapy by inducing arrhythmias and heart failure. Currently, many measures to prevent and treat MIRI are still difficult to apply in the clinical setting due to various reasons. Therefore, the investigation of the pathophysiological process and molecular mechanism of MIRI, as well as the search and discovery of new therapeutic strategies and targets is still a hot topic of research in the field of ischemic heart disease.

Oxidative stress is one of the most important pathological mechanisms in reperfusion injury, causing apoptosis, autophagy, inflammation, and some other damage in endothelial cells and cardiomyocytes through multiple pathways, resulting in irreversible cardiac injury and cardiac dysfunction [36]. Apoptosis, a unique form of gene-regulated cell death, has been shown to be primarily induced or accelerated during reperfusion or reoxygenation [37, 38]. Apoptosis has been suggested to cause infarct expansion [39], cardiac dysfunction, and even heart failure [40]. Increased levels of ROS have been shown to cause inflammation, endothelial cell-cell interactions and calcium overload, leading to increased mitochondrial release of pro-apoptotic genes [41]. Mechanisms of apoptosis induced by ROS have been systematically described [42]. Oxidative stress is a complex pathological process that regulates the release of extracellular vesicles (EVs) and affects EVs cargo loading. These EVs are taken up by the recipient cells and their released cargoes can regulate the redox status of target cells, affecting their function and potentially having deleterious effects on target cells[43–45]. Our previous studies have shown that hypoxia/reoxygenation (HR) induces oxidative stress and apoptosis in HUVECs [22]. In this study, we found that EMVs released from hypoxia-stressed HUVECs have an injurious effect on the heart under oxidative stress.

Changes in the physiopathological state of endothelial cells may affect the release of endothelial microvesicles (EMVs) and alter the cargo they carry. We collected supernatants from normal HUVECs, HR-injured HUVECs, DMSO post-treated HUVECs and propofol post-treated HUVECs after 24 h incubation to extract EMVs and performed whole transcriptome sequencing of EMVs. Analysis of the sequencing data revealed that the number of EMVs from hypoxic/reoxygenated HUVECs was significantly increased compared with those from normal HUVECs, and the lncRNAs carried by the two groups of EMVs were significantly different, which was consistent with the reported literature. The study[46]found that CD31^+^ EMVs (endothelium-derived microvesicles) in the blood of patients progressively increased with the progression of the coronary atherosclerotic process, with the highest expression in patients with acute myocardial infarction, strongly suggesting that endothelium-derived microvesicles are associated with myocardial damage. It has also been newly revealed by a study[47]that MIRI induces the release of extracellular vesicles (IR-EVs) from cardiac tissue and that IR-EVs induce apoptosis in cardiac myocytes, exacerbating cardiac injury and promoting local and systemic inflammation. In *vivo* and in *vitro* experiments in this study also confirmed that microvesicles from HR-injured HUVECs (HR-EMVs) exacerbated cardiac IR injury, induced oxidative stress and apoptosis in cardiomyocytes, and increased the infarct size.

Propofol is an intravenous anesthetic with antioxidant activity, which has been shown to protect organs damaged by oxidative stress [48, 49]. Our previous study confirmed that propofol attenuated HR-induced oxidative stress and autophagy in HUVECs and clarified that 100 µM is the optimal concentration of propofol to inhibit endothelial cell injury[21, 22]. Therefore, microvesicles derived from propofol post-treated HUVECs (HR + P)-EMVs may carry potent mediators against oxidative stress and apoptosis. In the present study, we observed that propofol treatment of endothelial cells attenuated the myocardial damaging effects of HR-EMVs. Compared with HR-EMVs and (HR + D)-EMVs, (HR + P)-EMVs significantly attenuated the oxidative stress and apoptotic effects on IR-injured cardiomyocytes, reduced myocardial infarct size and improved cardiac function.

Long non-coding RNA (lncRNA) is a non-coding RNA of > 200 nt in length that has been implicated in the development and progression of cardiovascular disease (CVD). In recent years, lncRNA derived from extracellular vesicles have been increasingly studied in cardiovascular diseases, providing new insights for the treatment of CVD[50, 51]. In this study, we analyzed the expression of lncRNAs in each group of EMVs by whole-transcriptome sequencing, and obtained 96 up-regulated lncRNAs and 71 down-regulated lncRNAs in (HR + P)-EMVs compared with (HR + D)-EMVs. We identified lncCCT4-2 as a potential effector molecule of (HR + P)-EMVs against cardiomyocyte injury among the up-regulated lncRNAs by combining differential ploidy, expression abundance and qPCR assay results. We observed that lncCCT4-2 expression was low in HR-injured AC16 cells, whereas it was significantly increased in AC16 cells co-cultured with (HR + P)-EMVs, suggesting that (HR + P)-EMVs containing lncCCT4-2 were internalized by AC16 cells and a large amount of lncCCT4-2 entered AC16 cells and could be stably expressed. We knocked down lncCCT4-2 in HUVECs by lentivirus followed by post-treatment with propofol to reduce the abundance of lncCCT4-2 expression in (HR + P)-EMVs. In AC16 cells co-cultured with sh-lncCCT4-2-(HR + P)-EMVs, the expression of lncCCT4-2 was significantly reduced, while sh-lncCCT4-2-(HR + P)-EMVs recapitulated a similar myocardial injurious effect as HR-EMVs and promoted oxidative stress and apoptosis in AC16 cells, suggesting that lncCCT4-2 is an effector of (HR + P)-EMVs against cardiomyocyte injury.

The Lncipedia database indicates that lncCCT4-2 is an antisense lncRNA for CCT4, which is a potential cis-regulatory coding gene for lncCCT4-2 and is usually the closest coding gene to lncCCT4-2, which is predicted to bind to CCT4 targets. Antisense lncRNAs can increase the stability of their sense mRNAs and promote the expression of sense mRNAs[52, 53]. In this study, we found that CCT4 mRNA and CCT4 protein were significantly increased in AC16 cells co-cultured with (HR + P)-EMVs, whereas CCT4 mRNA and protein levels were decreased in AC16 cells co-cultured with sh-lncCCT4-2-(HR + P)-EMVs, which was consistent with the expression trend of lncCCT4-2. RNA stability assays showed that overexpression of lncCCT4-2 prolonged the half-life of CCT4 mRNA, suggesting that lncCCT4-2 increased CCT4 protein expression by increasing the stability of CCT4 mRNA. However, the specific regulatory relationship between lncCCT4-2 and CCT4 is unclear and requires further.

CCT4 is a subunit of TRiC that has been implicated in cell proliferation, apoptosis and organ development [31–33], but the effect of CCT4 on cardiovascular disease remains unclear. We observed that knockdown of CCT4 caused (HR + P)-EMVs to show increased oxidative stress and apoptosis in HR-injured cardiomyocytes. Overexpression of CCT4 abolished the exacerbating effect of (HR + P)-EMVs silencing lncCCT4-2 on cardiomyocyte injury. Our study demonstrates that lncCCT4-2 derived from (HR + P)-EMVs mediates CCT4 inhibition of HR-EMVs-induced oxidative stress and apoptosis in cardiomyocytes.

Myocardial ischemia and reperfusion are common in cardiovascular surgery, and MIRI frequently occurs during surgery [54, 55]. Injury to vascular endothelial cells is the first critical step in the pathogenesis of myocardial IRI [7]. It is widely accepted that oxygen radicals have a significant negative impact on cell integrity during ischemia and reperfusion [36]. Thus, antioxidant anesthetics may help prevent or attenuate vascular endothelial cell and cardiomyocyte injury during cardiovascular surgery. Based on the current study, propofol alters the deleterious biological components of EMVs during IR and blocks the spread of harmful mediators between endothelial cells and cardiomyocytes. Thus, intravenous propofol during the perioperative period helps to reduce intraoperative or postoperative cardiac IRI.

The advantages of extracellular vesicles (EVs) for cardiac-targeted delivery of proteins, mRNAs, non-coding RNAs and available drugs have the potential to become therapeutic approaches for cardiovascular disease[56]. Engineering modifications of EVs facilitate the translation of basic research on EVs into clinical applications. For example, recent literature reports that loading of si-RCP into MSC-derived EVs (MSC-EVs/si-RCP) by electroporation technique resulted in enhanced repair of degenerated discs by MSC-EVs under hypoxia[57]. Our study provides new insights into the specific mechanism by which propofol inhibits cardiac IRI: (HR + P)-EMVs increased lncCCT4-2 expression in cardiomyocytes, which in turn modulated CCT4 mRNA stability and promoted CCT4 protein expression, thereby inhibiting oxidative stress and apoptosis in IR-injured cardiomyocytes. Thus, both lncCCT4-2 and CCT4 are promising targets for the development of cardioprotective agents, and genetic modification of EMVs donor cells or loading of OE-lncCCT4-2 into EMVs may provide new ideas for the treatment of MIRI.

This study also has some limitations. In this study, lncCCT4-2 was the key molecule in (HR + P)-EMVsd against myocardial injury, while whether other bioactive components in (HR + P)-EMVs have myocardial protective effects needs further investigation. We suggested that the anti-apoptotic effect of lncCCT4-2 from (HR + P)-EMVs is attributed to its mediated upregulation of CCT4 expression, but the exact mechanism needs further investigation.

## Conclusions

In summary, HR-EMVs exacerbated oxidative stress and apoptosis in cardiomyocytes subjected to ischemia-reperfusion, whereas the damaging effect of (HR + P)-EMVs on IR-injured cardiomyocytes was significantly attenuated compared with HR-EMVs. We revealed the underlying mechanism: the lncCCT4-2 from (HR + P)-EMVs increased the stability of CCT4 mRNA and the level of CCT4 protein in cardiomyocytes, thereby inhibiting oxidative stress and apoptosis in cardiomyocytes. Our study provides a new direction for the treatment of cardiac IRI.

## Materials and methods

### Cell culture and experiment grouping

The parent cells of endothelial microvesicles (EMVs) are HUVECs and the recipient cells are AC16 cells. HUVECs (Cell Bank of Chinese Academy of Sciences) and AC16 cells (Shanghai Institute of Cell Biology) were cultured in DMEM supplemented with 10% fetal bovine serum and 1% penicillin-streptomycin at 37 °C with air containing 5% CO2. The cell culture medium was regularly replaced in a 24 h cycle. HUVECs at 70% confluence were exposed to hypoxia-reoxygenation (HR). The hypoxic challenge was achieved with a gas mixture containing 1% O2, 5% CO2, and 94% N2 for 12 h in a hypoxic chamber (Billups-Rothenberg, Del Mar, CA, USA) and then reoxygenated in the DMEM containing 10% EVs-free FBS.

Reoxygenated HUVECs were first treated with or without propofol at concentrations of 25 μm, 50 μm, 100 μm, and 150 μm for 24 h. Cultures were then collected to obtain HR-EMVs, (HR + P25)-EMVs, (HR + P50)-EMVs, (HR + P100)-EMVs, and (HR + P150)-EMVs, respectively. The above EMVs were co-cultured with hypoxic/reoxygenated AC16 cells for 24 h, and then 100 μm was clarified as the optimal concentration for propofol treatment based on CCK-8 assay and apoptosis flow cytometry. Therefore, (HR + P)-EMVs and (HR + D)-EMVs used in subsequent experiments refer to EMVs derived from HUVECs post-treated with 100 μm propofol or an equivalent volume of DMSO.

AC16 cells, as receptor cells for EMVs, were divided into five groups: normal control group (NC), hypoxia-reoxygenation group (HR), HR-EMVs treatment group (HR + HR-EMVs), (HR + D)-EMVs treatment group (HR+(HR + D)-EMVs) and (HR + P)-EMVs treatment group (HR+(HR + P)-EMVs). AC16 cells were first hypoxic for 8 h and then co-cultured with the above EMVs (10^9 particles/ml) under reoxygenation conditions for 24 h, followed by a series of biological experiments.

### Animals and experiment grouping

Animal studies were reviewed and approved by the Experimental Animal Ethics Committee of Guangdong Medical University (ID number: GDY18010127.1). 110 male C57BL/6 mice, 6–8 weeks old, weighing (24 ± 2) g, were purchased from the Experimental Animal Center of Southern Medical University (Guangdong, China) and housed at the Experimental Animal Center of the Affiliated Hospital of Guangdong Medical University (license number: SYXK (Guangdong) 2020 − 0147). Mice were housed in a specific pathogen-free (SPF) room with constant temperature (24 ± 1 °C), humidity (60-70%) and light (12 h light-dark cycle). All mice were fed ad libitum and housed for at least 1 week prior to the experiment to reduce environmental stress. Mice used in this study were euthanized by cervical dislocation.

In this study, 30 mice were first randomly divided into 6 groups: sham + PBS, sham + HR-EMVs, sham+(HR + P)-EMVs, IR + PBS, IR + HR-EMVs and IR+(HR + P)-EMVs, 5 mice in each group, and then echocardiography and plasma LDH assays were performed, and it was found that HR-EMVs and (HR + P)-EMVs had no significant effect on the cardiac function of normal mice. Then 80 mice were randomly divided into 4 groups: sham + PBS, IR + PBS, IR + HR-EMVs and IR+(HR + P)-EMVs, 20 mice in each group, and the hearts were extracted for TTC staining, TUNEL staining, DHE staining and Western Blot.

### Myocardial IR model

Mice were anesthetized by inhalation with 4-6% isoflurane, then intubated with a 1.5 mm catheter, and connected to a ventilator with the following parameters: tidal volume 1.8-2 mL/time, respiratory rate 110–120 breaths/min, and respiratory ratio 1.3:1. Under mechanical ventilation, the chest was opened and the 3/4 rib space was dilated, and the mouse heart was fully exposed by tearing the pericardium under direct vision. Ischemia-reperfusion (IR) group: 2 mm from the lower edge of the left auricle of the heart, 1/3 thickness of the myocardial layer was penetrated with an 8 − 0 suture, followed by ligation of the left anterior descending branch of the coronary artery with a live knot. A whitening of the distal left ventricle and an elevated ST segment in ECG lead II were observed, indicating successful ischemia. After 45 min of myocardial ischemia, the heart was exposed again, and three regions at the edge of myocardial infarction were selected for injection of 50 µL of HR-EMVs, (HR + P)-EMVs (2 × 10^9/µL), or equal volume of PBS, and then the live knot was loosened to restore myocardial blood supply. The observation of ST-segment regression on ECG suggested successful reperfusion. Sham group was operated the same as IR group except that the coronary artery was not ligated. The chest cavity was closed after venting by gently squeezing it. After performing reperfusion for 3 days, the hearts of mice were removed for subsequent experiments.

### Sequencing and analysis of EMVs-derived lncRNAs

Supernatants were collected from the following three groups of HUVECs cells: hypoxic/reoxygenated (HR) HUVECs, DMSO-post-treated (HR + D) HUVECs and propofol-post-treated (HR + P) HUVECs, and 50 ml of supernatant was collected from each group of cells, and three replicates were collected. The cell supernatants of the three groups of HUVECs were sent to Guangzhou Geneseed Biotech Co., Ltd. (http://www.geneseed.com.cn/) for whole transcriptome sequencing and data analysis of endothelial microvesicles (EMVs). HR-EMVs, (HR + D)-EMVs and (HR + P)-EMVs were extracted by ultra-high speed centrifugation, and then total RNA was extracted by Trizol method. 1-10ng RNA was taken for library construction using the Clontech SMARTer Stranded Total RNA seq Kit, and finally sequenced using the Novaseq 6000 PE150 mode. Transcripts of long-stranded non-coding RNAs (lncRNAs) were analyzed using the LNcipedia (https://lncipedia.org/) database. The filtered lncRNAs were subjected to differential expression analysis (|log2(FC)|> 1 (FC: fold-change), P < 0.05, FDR < 1), and finally 96 and 71 transcripts of up- and down-regulated lncRNAs were obtained. Among the upregulated lncRNAs, we screened five lncRNAs with significant differential expression and high expression abundance for qPCR validation, and finally identified lncCCT4-2 as a potential effector molecule of (HR + P)-EMVs against myocardial injury.

### Data analysis and statistics

Cells cultured in the well plate were randomly used in different experimental groups. At least 3 biologically independent experiments were performed for in vitro cell experiments, and at least 5 biologically independent experiments were performed for in vivo animal experiments. Experimental data are expressed as mean ± SEM. Representative morphological images were taken from at least three biologically independent experiments with similar results. The unpaired two-tailed Student t-test was used for comparisons between two groups, and one-way ANOVAs followed by Bonferroni’s post hoc test was used for multi-component comparisons. All data were analysed with SPSS software (version 19.0) using the appropriate statistical analysis methods as indicated in the figure legends. Significance was accepted at *P < 0.05, **P < 0.01, ***P < 0.001, ns, not significant.

## Electronic supplementary material

Below is the link to the electronic supplementary material.

Additional file 1:
Supplementary Figure 1 Normoxic AC16 cells were co-cultured with HR-EMVs at different concentrations (10^7, 10^8 and 10^9 particles/ml) for 24 h: (A) CCK-8 assay for AC16 cell viability. (B) Flow cytometry detection of apoptotic rate in AC16 cells after annexin V-FITC/PI double staining. (C) quantitative analysis of apoptotic cells. HR-injured AC16 cells were co-cultured with HR-EMVs, (HR + P25)-EMVs, (HR + P50)-EMVs, (HR + P100)-EMVs, and (HR + P150)-EMVs for 24 h, respectively: (D) CCK-8 assay for AC16 cell viability. (E) Flow cytometry detection of apoptotic rate in AC16 cells after annexin V-FITC/PI double staining. (F) quantitative analysis of apoptotic cells. These data were representative results (n = 3) of three repetitions. *P < 0.05, **P < 0.01, ***P < 0.001, ns, not significant. Data are expressed as mean ± SEM.
Supplementary Figure 2 (A) HUVECs were infected with LV-sh-lncCCT4 or LV-sh-NC for 72 h. The knockdown efficiency of lncCCT4-2 in HUVECs was detected by RT-qPCR. Transfection of AC16 cells with sh-CCT4 and OE-CCT4 plasmids for 48 h: (B-C) Detection of CCT4 mRNA expression in AC16 cells by RT-qPCR. (D-E) Western blotting analysis of CCT4 protein expression in AC16 cells to detect the transfection efficiency of sh-CCT4 and OE-CCT4 plasmids. These data were representative results (n = 3) of three repetitions. *P < 0.05, **P < 0.01, ***P < 0.001, ns, not significant. Data are expressed as mean ± SEM.
Supplementary 3 The genomic location and sequence of lncCCT4-2 and CCT4.
Supplementary 4 Prediction of lncCCT4-2 and CCT4 mRNA binding sites.

Additional file 2: Supplementary materials and methods.

## Data Availability

The data supporting this study’s findings are available from the corresponding authors upon reasonable request.

## Abbreviations

IR: ischemia-reperfusion
MIRI: myocardial ischaemia-reperfusion injury
MVs: microvesicles
HR: hypoxia/reoxygenation
HUVECs: human umbilical vein endothelial cells
EMVs: endothelial microvesicles
DMSO: dimethyl-sulfoxide
RNA-Seq: RNA sequencing
LncRNA: long non-coding RNA
CCT4: chaperonin containing t-complex 4
ROS: reactive oxygen species
IHD: ischemic heart disease
EVs: extracellular vesicles
EEVs: endothelial extracellular vesicles
CCK-8: cell counting kit-8
LDH: lactate dehydrogenase
TEM: transmission electron microscopy
NTA: nanoparticle tracer analyzer
DCFH-DA: Dichloro-dihydro-fluorescein diacetate
PI: propidium iodide
RT-qPCR: Real-time quantitative polymerase chain reaction
LVEF: left ventricular ejection fraction
LVFS: left ventricular fractional shortening
