# Supplementary material for: Propofol inhibits myocardial injury induced by microvesicles derived from hypoxia-reoxygenated endothelial cells via lncCCT4-2/CCT4 signaling
Source: Biol Res. 2023 May 5;56:20. doi: 10.1186/s40659-023-00428-3 (PMC10161458; doi:10.1186/s40659-023-00428-3)

**Supplementary Figure 1**

**
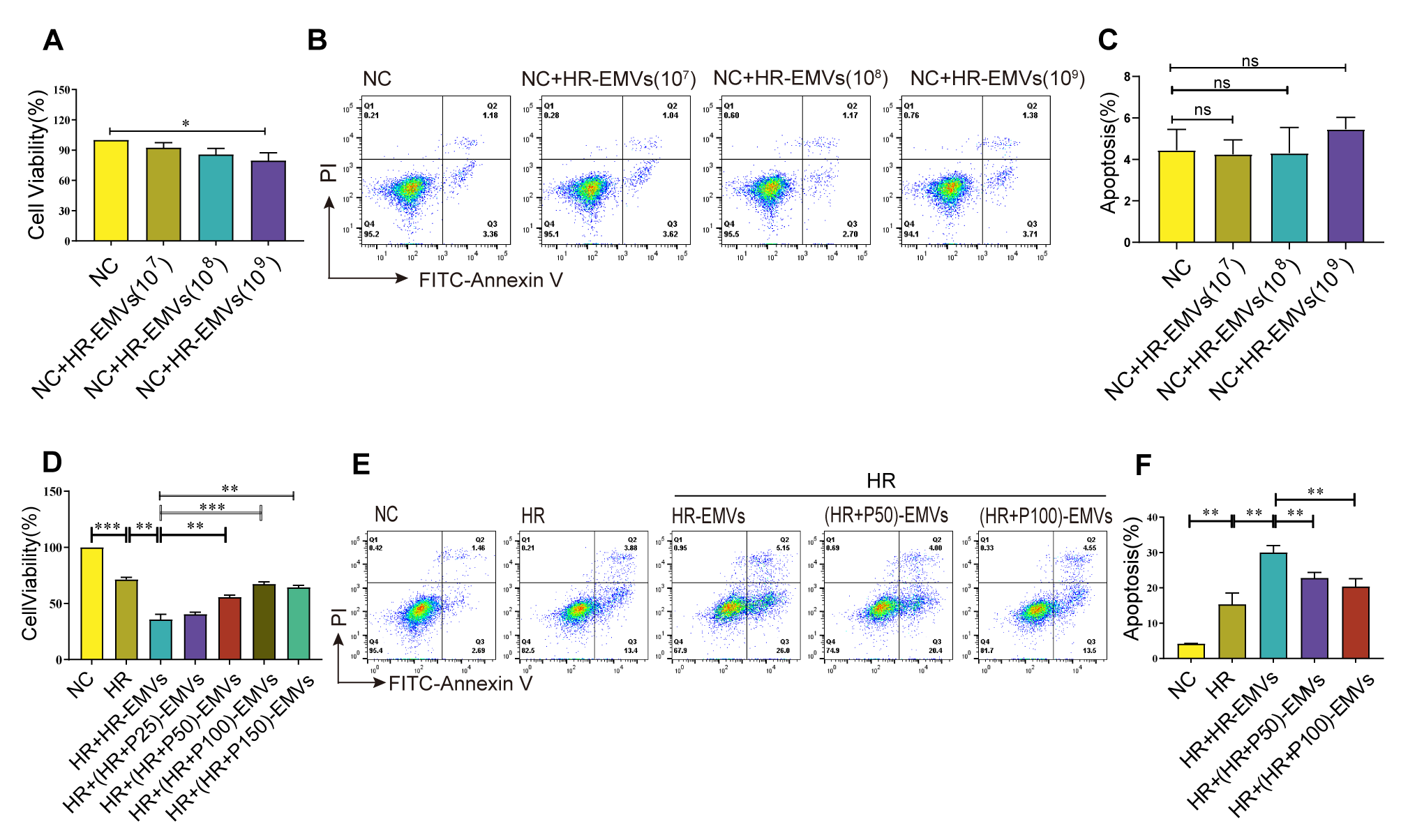
**

**Fig. S1: *(HR+P)-EMVs showed less effects on inducing oxidative stress and apoptosis compared with HR-EMVs in hypoxia-reoxygenated cardiomyocytes***

Normoxic AC16 cells were co-cultured with HR-EMVs at different concentrations (10^7, 10^8 and 10^9 particles/ml) for 24 h: **(A)** CCK-8 assay for AC16 cell viability. **(B)** Flow cytometry detection of apoptotic rate in AC16 cells after Annexin V-FITC/PI double staining. **(C)** Quantitative analysis of apoptotic cells. HR-injured AC16 cells were co-cultured with HR-EMVs, (HR+P25)-EMVs, (HR+P50)-EMVs, (HR+P100)-EMVs, and (HR+P150)-EMVs for 24 h, respectively: **(D)** CCK-8 assay for AC16 cell viability. **(E)** Flow cytometry detection of apoptotic rate in AC16 cells after Annexin V-FITC/PI double staining. **(F)** Quantitative analysis of apoptotic cells. These data were representative results (n = 3) of three repetitions. *P < 0.05, **P < 0.01, ***P < 0.001, ns, not significant. Data are expressed as mean ± SEM.

**Supplementary Figure 2**

**
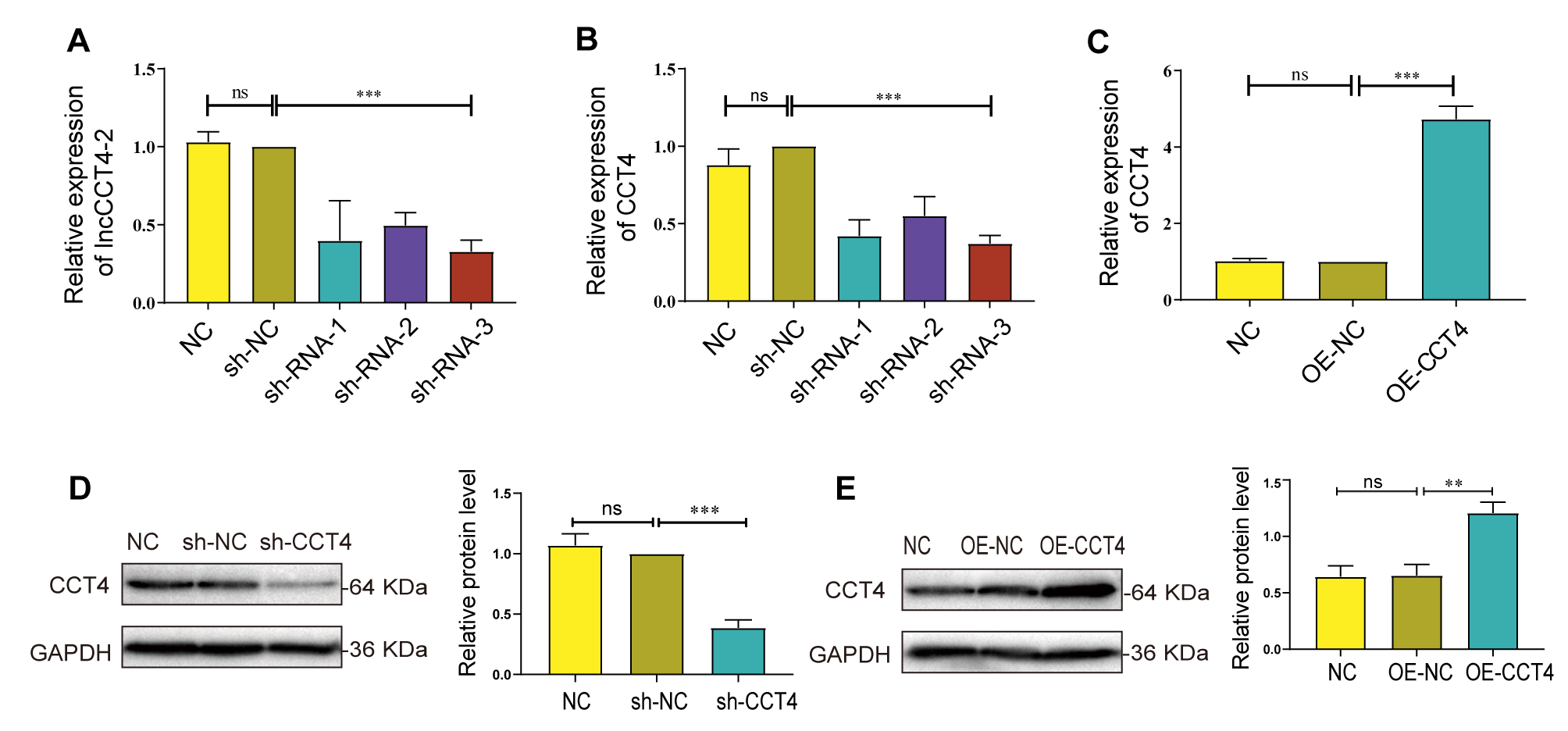
**

**(A)** HUVECs were infected with LV-sh-lncCCT4 or LV-sh-NC for 72 h. The knockdown efficiency of lncCCT4-2 in HUVECs was detected by RT-qPCR. Transfection of AC16 cells with sh-CCT4 and OE-CCT4 plasmids for 48 h: **(B-C)** Detection of CCT4 mRNA expression in AC16 cells by RT-qPCR. **(D-E)** Western blotting analysis of CCT4 protein expression in AC16 cells to detect the transfection efficiency of sh-CCT4 and OE-CCT4 plasmids. These data were representative results (n = 3) of three repetitions. *P < 0.05, **P < 0.01, ***P < 0.001, ns, not significant. Data are expressed as mean ± SEM.

**Supplementary 3: The genomic location and sequence of lncCCT4-2 and CCT4**


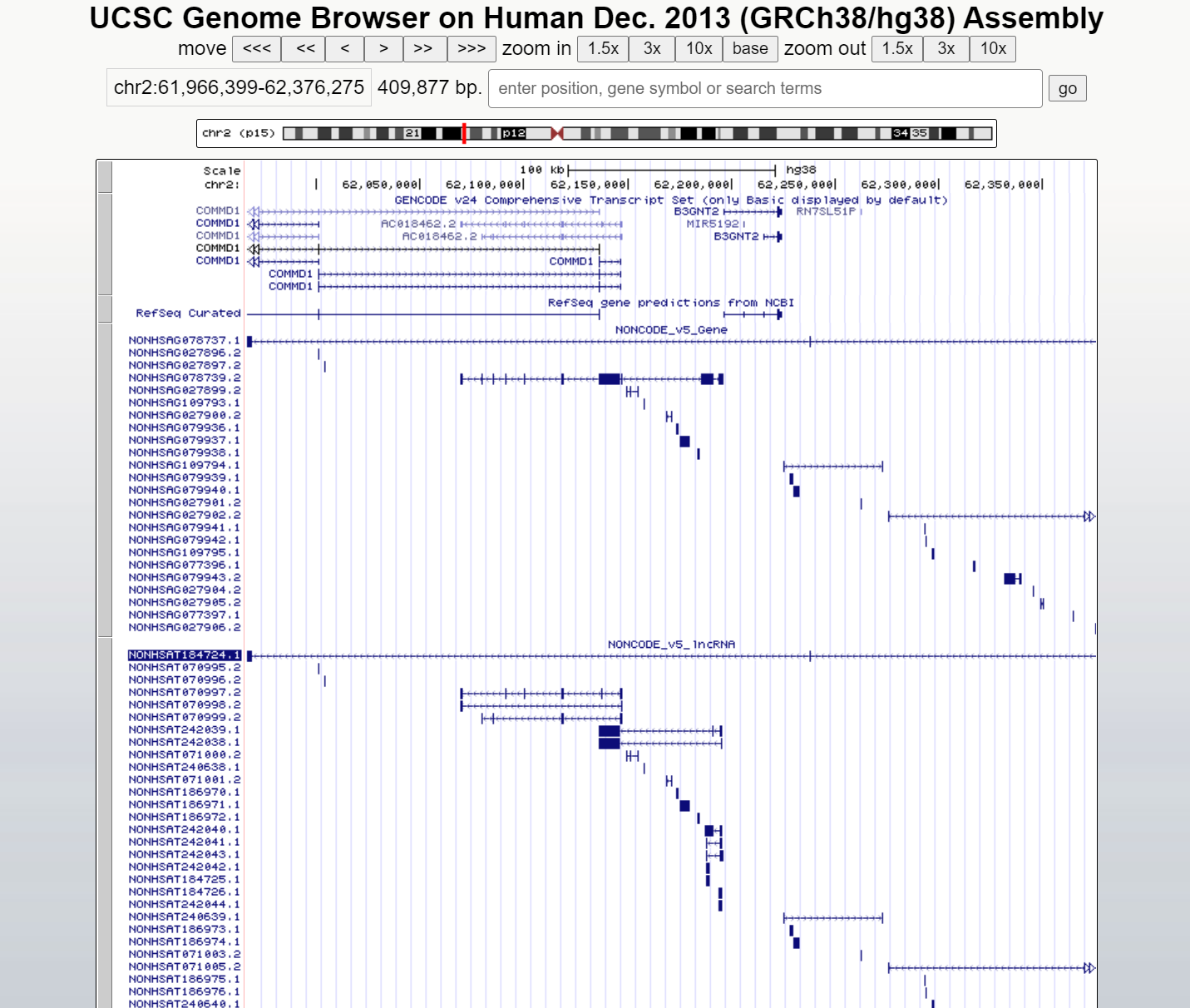


**Sequence of lncCCT4-2, chr2:(61966400-62376275) 2328 bp**

GAGACAGAGTCTCACTGTGTCACCCAGGCTGGAGTGCAGTAGCACAATCTTGGCTCACTGCAACCTCCACCTCCCGGGTTCAGGTGGTTCTCCTGCCTCAGCCTCCTGAGTAGCTGGGATTACAGGCACCCACCACCACGCCTGGCTAATTTTTGTATGTTTAGTATAGACAGGGTTTCACCATGTTGGCCAGGCTGGTCTCGAACTCCTGACCTCAGATGATCCACCCACCTCAGCCTCCCAAAGTGCTGGGATTACAGGCAAATGGACTGCTTGAGCTCAGGAATTTGAAACCAGCCTGGGCAACATGGAGAAATCTCAACTATATAAAAAATACAAAAAATGAGCCAGGCATGGTGGCATGCACCTGTGGTGACAACTACTGGAAAGGCTGAGGTAGGAGGATCACTTGAGCCCAGAAGGCCAAGGCTGCAGTGAGCCAAAATTATACCATGGGACTCCAGCCCAGCAACAGAGTGATACCCTCCCTCCCTCCCCTCCATCAAGAAAAGGAAAATGGTCCAAAACTCTAAGTAGATATAGTCCAAAAAGAAAAGCAAACTGATTTTGTCCTTTCTTTTCACCTTCAGTAAATTTGTGAAATGTGATTTACTGTACAATTAATTATAGTATGGTTTTACTGTTTCAAATGTATTCTCTGGGCTTCAATTTCCCTAAAATTAAGCAAAGAGAATTTGCTATATGAGCTGCATGTGCCCCCAAGTAATTACATTCCATATTGCTATATCTGTAGGTTGAGCCATATAAATACAACAGTATTCAACTGTTTTGGACTCACAAGTATGCTAATTTCAAATGGTTCAACCTTACTAAATCTCTTAAATTTTATTTTCCCCTATAGACTATGTTTGGCACCAAGCGGCTTTTGTTTTCTTTTTTTTTTCTGAGAGGGAGTCTCACTGTGTCACCCAGGCAGGAGTGCAGTGGCACAATCTTGGCTCACTGCAACCTCTGCCTCCTGGGTTCAAGCAGTTCTCCTGCCTCAGCCTCCCAAGTAGCTGGGATTACAGGCACACGCCACCACGCCCGGCTAATTTTTGTATTTTTAGTAGAGATAGGGTTTCATTGTGTTGGCCAGGCTGGTCTCGAACTCCTGACCTCAGATGATCTGCCAGCCTTGGCCTTCCAAAGTGCTGGGATTATAGGCATGAGCCACCACACCCAGCCTATTTTCTTAAAATAGAGCAAAATGTTTAACATTATTATGAAAGCCAGTGATGAGAGCCAGTTCTCAATTGACATTCAGGACTGAATACACTTTATCAGACATTTTTTAAAGCAAAGAAAAAGATGATACTCTGATATGGAAACCCTCCTAAATTACTATTAATATTTCACAATTCATATTTTGGAATACTCGCAGAATTTGTACTAGCCCATTCAGCTTTATGATCCCACTTATTATCAGCTTATAGTCTGTGGCCTAAGTTATAGTTTTCACCACCTGCCTACTATGTATCAGGCCCTGTCCCAATCCTTCAAATAAGATGAACAGCATATGGACCTGCTTTTGAAGAGCTTTCAATCTGGTTATGTAAACAAGAGCTCAACATCCTTGGCTTTCCTGATTTGCTAGAAAATCTAGAGCAATGCTACCCAAAGTATGGTCTACATATCAGTGCCAGACCACAAACTTCACTACCAGTCCACCATGAGGTAATTATACAAACTGAAAGTAAATATTTAGAAACTTTCATGGAAAAATGACTATTTTTATGTCAATTAACCTAATAAAATAATCGGATTGTATGTTTTTATGTATTTTTGGTGAGGAATTGAACATTTTTTTAAAAAACAAAACTGGTCCTCAACAGACTGCTTCTTAAATGTAAGAAGCACTGCTGTGAAGGTAGACAGATTGGACAGTTCTGCTATCTTTGCAACTTAGCTCTTCTGCATTTGAAAGTTAAAATATTCGTTTCATATTAGACTTCATCAACTGGCCCCATTTCCACTATAATAAAGATCCTGGTTACATTTGATAAGGGAAAAAGGACACCAAGAAGAAAATAAGATGCTGGCTATGGAAGTCAGGAAGATAAAAGTTACTAAAAATAAGAAAAGAGAAAAAATGTTACTGCTCTTCATGGAAGAGAAAGGAAAATACTTCAAGTTGACGGTAAATTTTTAAAAACCACTGTTGTGTATGCCCGGGTCTTCTTAGATAAATAGAAGTGAAATGAAATACATTTTAAAGGCAAATTCATATTTCCTATTATTAGATTTAATATACAACCAACTAGTGTTTCAATTGGGTATTACTGCCATATTCTGCATGCTAATTTAACAAATTTACATATTTTAAAA

**Sequence of CCT4 mRNA, chr2: (61,868,085-61,888,656) 2376 bp**

CTTCTCCGCCTCCGCCTCCTCCCGACGCCGGCGCCGCTTTCTGGAAGGTTCGTGAAGGCAGTGAGGGCTTACCGTTATTACACTGCGGCCGGCCAGAATCCGGGTCCATCCGTCCTTCCCGAGCCAACCCAGACACAGCGGAGTTTGCCATGCCCGAGAATGTGGCACCCCGGAGCGGGGCGACTGCCGGGGCTGCCGGCGGCCGCGGGAAAGGCGCCTATCAGGACCGCGACAAGCCAGCCCAGATCCGCTTCAGCAACATTTCCGCCGCCAAAGCGGTTGCTGATGCTATTAGAACAAGCCTTGGACCAAAAGGAATGGATAAAATGATTCAAGATGGAAAAGGTGATGTAACCATTACAAATGATGGTGCTACCATTCTGAAACAAATGCAAGTATTACATCCAGCAGCCAGAATGCTGGTGGAGCTGTCTAAGGCTCAAGATATAGAAGCAGGAGATGGCACCACATCAGTAGTCATCATTGCTGGCTCCCTCTTAGATTCTTGTACCAAGCTTCTTCAGAAAGGGATTCATCCAACCATCATTTCTGAGTCATTCCAGAAGGCCCTGGAAAAGGGCATTGAAATCTTGACTGACATGTCTCGACCTGTGGAACTGAGTGACAGAGAAACTTTGTTAAATAGTGCAACCACTTCACTGAACTCAAAGGTGGTTTCTCAGTATTCAAGTCTGCTTTCTCCAATGAGTGTAAATGCAGTGATGAAAGTGATTGACCCAGCCACAGCCACCAGTGTAGATCTTAGAGATATTAAAATAGTTAAGAAGCTTGGTGGGACAATTGATGACTGTGAGTTGGTGGAAGGGCTGGTTCTCACCCAAAAAGTGTCAAATTCTGGCATAACCAGAGTTGAAAAGGCCAAGATTGGGCTTATTCAGTTTTGCTTATCTGCTCCCAAAACAGACATGGATAATCAAATAGTGGTTTCTGACTATGCCCAGATGGACCGAGTGCTGCGAGAAGAGAGAGCCTATATTTTAAATTTAGTGAAGCAAATTAAAAAAACAGGATGTAATGTCCTTCTCATACAGAAATCTATTCTAAGAGATGCTCTTAGTGATCTTGCATTACACTTTCTGAATAAAATGAAGATCATGGTGATTAAGGATATTGAAAGAGAAGACATTGAATTCATTTGTAAGACAATTGGAACCAAGCCAGTTGCTCATATTGACCAATTTACTGCTGACATGCTGGGTTCTGCTGAGTTAGCTGAGGAGGTCAATTTAAATGGTTCTGGCAAACTGCTCAAGATTACAGGCTGTGCCAGCCCTGGAAAAACAGTTACAATTGTTGTTCGTGGTTCTAACAAACTGGTGATTGAAGAAGCTGAGCGCTCCATTCATGATGCCCTATGTGTTATTCGTTGTTTAGTGAAGAAGAGGGCTCTTATTGCAGGAGGTGGTGCTCCAGAAATAGAGTTGGCCCTACGATTAACTGAATATTCACGAACACTGAGTGGTATGGAATCCTACTGCGTTCGTGCTTTTGCAGATGCTATGGAGGTCATTCCATCTACACTAGCTGAAAATGCCGGCCTGAATCCCATTTCTACAGTAACAGAACTAAGAAACCGGCATGCCCAGGGAGAAAAAACTGCAGGCATTAATGTCCGAAAGGGTGGTATTTCCAACATTTTGGAGGAACTGGTTGTCCAGCCTCTGTTGGTATCAGTCAGTGCTCTGACTCTTGCAACTGAAACTGTTCGGAGCATTCTGAAAATAGATGATGTGGTAAACACTCGATAATCTGGATAACTGACTAGCACCATTATGATCACCAGTATTGTGGCTGGAATGGAAGAAGATCACCTTGGTGTTCCTTGTTTGGAAGATTATTTCCTCTGAATTTCTGGGCTTGGTCTTCCAGTTGGCATTTGCCTGAAGTTGTATTGAAACAATTTAATGAAAATATTAAATATTTGGTTTCAAAAGGCAGATTTATCTTCTCCCAACATTCTGTTATTTCTGATACTTTTGAAAAACTAATAAAAACTAATAAAAGAAGCGTAAAAAGTGAGTTTACATGTTGAGGAAAAAAATGGCCCAATATGCTCATCACTGATAAATGCTCCCTGGCCTTAAAAACTACCAACATATAATATATATGCTGTCTTAAAAGTTAATGATCCAAGTGGCACCTCTCTGAACCTACTTTGGCTTGGGAGGCTGCCCAGTTAAAACAAAAATAAGTTAATGGTACAGAAAGAGAATGAAAAATGAAAGCCTCCTTTTATCCTATCATCCTAATTCCTTTTCCCAGTAATAATGACTGCTGTGTTGGATTCCTTCTGCAAATAAAAGTGTATACATATATGTAGCAAATCTTACTTAAACAAAAGGGTTTCTTAACTAA

**Supplementary 4: Prediction of lncCCT4-2 and CCT4 mRNA binding sites**

**<http://rna.informatik.uni-freiburg.de/IntaRNA/Result.jsp?toolName=IntaRNA&jobID=6251481>**


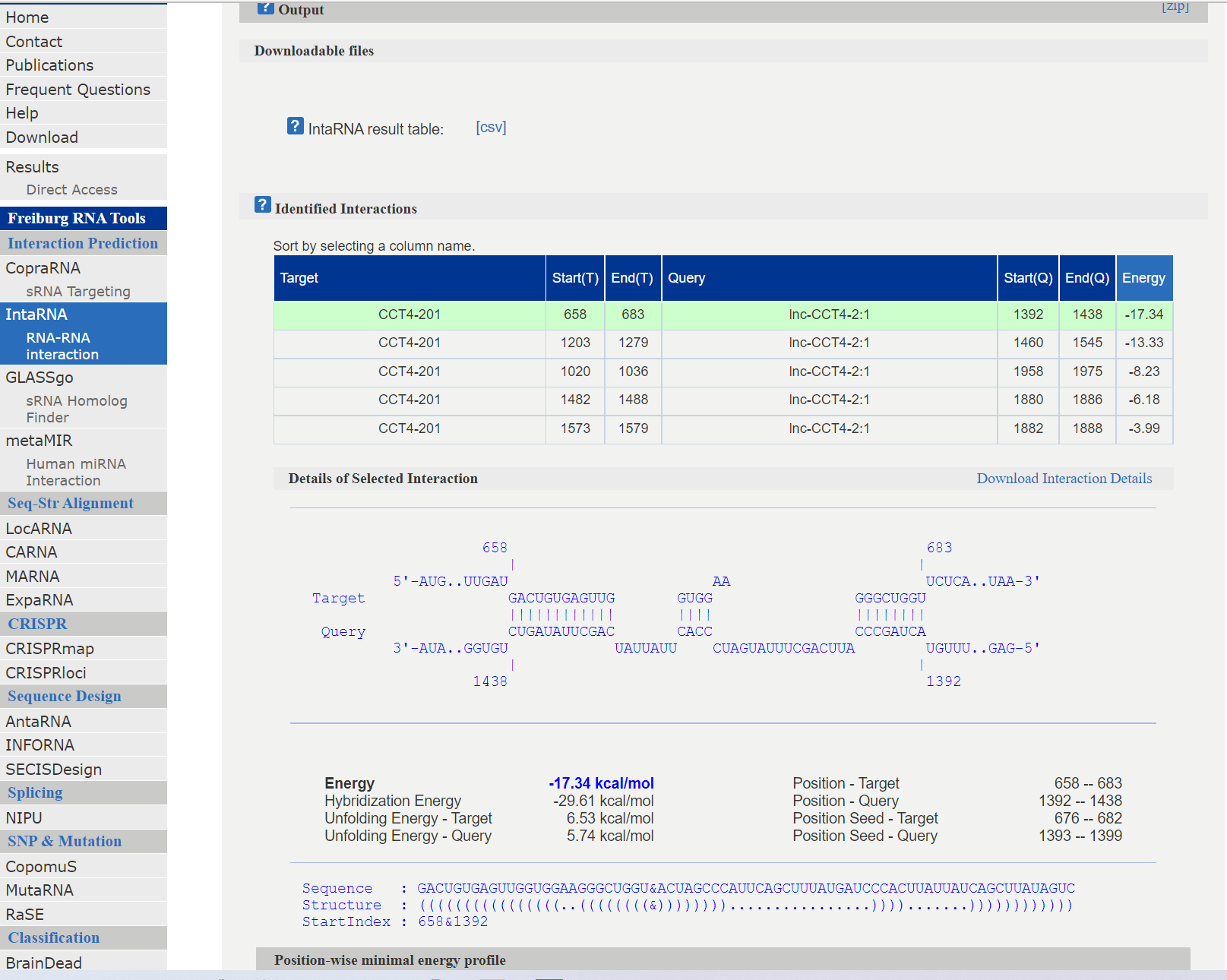

Supplement: Supplementary file 1 — Additional file 1: Supplementary Figure 1 Normoxic AC16 cells were co-cultured with HR-EMVs at different concentrations (10^7, 10^8 and 10^9 particles/ml) for 24 h: (A) CCK-8 assay for AC16 cell viability. (B) Flow cytometry detection of apoptotic rate in AC16 cells after annexin V-FITC/PI double staining. (C) quantitative analysis of apoptotic cells. HR-injured AC16 cells were co-cultured with HR-EMVs, (HR + P25)-EMVs, (HR + P50)-EMVs, (HR + P100)-EMVs, and (HR + P150)-EMVs for 24 h, respectively: (D) CCK-8 assay for AC16 cell viability. (E) Flow cytometry detection of apoptotic rate in AC16 cells after annexin V-FITC/PI double staining. (F) quantitative analysis of apoptotic cells. These data were representative results (n = 3) of three repetitions. *P < 0.05, **P < 0.01, ***P < 0.001, ns, not significant. Data are expressed as mean ± SEM. Supplementary Figure 2 (A) HUVECs were infected with LV-sh-lncCCT4 or LV-sh-NC for 72 h. The knockdown efficiency of lncCCT4-2 in HUVECs was detected by RT-qPCR. Transfection of AC16 cells with sh-CCT4 and OE-CCT4 plasmids for 48 h: (B-C) Detection of CCT4 mRNA expression in AC16 cells by RT-qPCR. (D-E) Western blotting analysis of CCT4 protein expression in AC16 cells to detect the transfection efficiency of sh-CCT4 and OE-CCT4 plasmids. These data were representative results (n = 3) of three repetitions. *P < 0.05, **P < 0.01, ***P < 0.001, ns, not significant. Data are expressed as mean ± SEM. Supplementary 3 The genomic location and sequence of lncCCT4-2 and CCT4. Supplementary 4 Prediction of lncCCT4-2 and CCT4 mRNA binding sites. [file 40659_2023_428_MOESM1_ESM.docx]
