# Supplementary material for: Propofol inhibits myocardial injury induced by microvesicles derived from hypoxia-reoxygenated endothelial cells via lncCCT4-2/CCT4 signaling
Source: Biol Res. 2023 May 5;56:20. doi: 10.1186/s40659-023-00428-3 (PMC10161458; doi:10.1186/s40659-023-00428-3)
Supplement: Supplementary file 2 — Additional file 2: Supplementary materials and methods. [file 40659_2023_428_MOESM2_ESM.docx]

**Supplementary materials and methods**

**Reagents and antibody**

Propofol solution was diluted with dimethyl sulfoxide (DMSO), and the final concentration of propofol in the HUVEC culture medium was maintained at 100uM, while DMSO was lower than 0.1%. The follows were the reagents used in our experiment: Dulbecco's modified eagle’s medium (DMEM), fetal bovine serum(FBS), penicillin-streptomycin (100×), trypsin-EDTA, Opti-MEM (all from GIBCO Laboratories, Grand Island, NewYork,USA), DMSO and phosphate buffer solution(PBS). The follows was the antibody we used for experiment: anti-Tubulin, anti-GAPDH, anti-Bax, anti-Bcl-2, anti-cleaved-caspase3, anti-CCT4, anti-CD105, anti-CD144. Assay kits used in the experiment were as follows: cell counting kit-8 (CCK-8), lactate dehydrogenase (LDH) assay kit, reactive oxygen species (ROS) kit, Annexin V-FITC apoptosis detection Kit, PKH26, one step TUNEL apoptosis assay kit, 2,3,5-Triphenyltetrazolium chloride, evans blue, the details of reagents and antibody mentioned above was available in supplementary data **(Table 1 and 2)**.

**Table 1. Information of antibodies used in the study**

| **Primary antibodies** | **Dilution** | **Clone/Catalog** | **Host species** | **Supplier** |
| --- | --- | --- | --- | --- |
| Cleaved Caspase-3 | 1:1000 | 9664S | Rabbit | Cell Signaling Technology, USA |
| Bax | 1:1000 | 2772S | Rabbit | Cell Signaling Technology, USA |
| Bcl-2 | 1:1000 | GR3239757-15 | Rabbit | Proteintech, China |
| CCT4 | 1:1000 | 21524-1-AP | Rabbit | Proteintech, China |
| Tubulin | 1:1000 | 11224-1-AP | Mouse | Proteintech, China |
| GAPDH | 1:1000 | AP0063 | Rabbit | Bioworld, China |

**Table 1. Cont.**

| **Second antibodies** | **Dilution** | **Clone/Catalog** | **Host species** | **Supplier** |
| --- | --- | --- | --- | --- |
| Goat anti-mouse IgG(H+L) | 1:5000 | 20000261 | Mouse | Proteintech,China |
|  |  |  |  |  |
| Goat anti-rabbit IgG(H+L) | 1:5000 | 20000311 | Rabbit | Proteintech, China |

**Table 2. The reagent and assay kit**

| **reagent and assay kit** | **Clone/Catalog** | **Supplier** |
| --- | --- | --- |
| Propofol | [D126608](https://www.sigmaaldrich.cn/CN/zh/product/aldrich/d126608) | Sigma-Aldrich, USA |
| [Cell-Counting-Kit-8](http://www.baidu.com/baidu.php?url=a00000KEJeCxDFezE_NsPdyeYHgNugM0rrfUCXu1DKfhNTFK_maFCeFm3EUk8PMRRlTpMouxFtsMZ3HGEIihexG32Zi_e1rVF7LHcYm3_CBfwDSLnVatKk7kwGjzni9ZrPvztXvN99ZgUTtXsP7NrC2FHIX7Q5qoQstrGC2urBXCFrr1bB1QZV2XhHY3SCrbBnDT7-mA0BzECcaF8Qj-VC3Pj0Y0.7Y_NR2Ar5Od663rj6tV2UCpblRqArunTxF0S2GqeljG_OpS9KahWw1s8swRZ4_OpS9MvTStTYQ7qvXdmhrMIu_oe2lSguz8HG4UVis1f_unhOmC.U1Yk0ZDqmvPornoREnAlY0Kspynqn0KsTv-MUWYvPHN9nvDYnHwBnA7-uhn1PW-9PhP-rAmkmWbkmHDdn6KY5yPWpzRzwjW58QelErzz3_oREnAlY0KGUHYznWR0u1dEugK1n0KdpHdBmy-bIykV0ZKGujYY0APGujYknH00UgfqnH0kPdtknjD4g1csPH7xnW0vn-t1PW0k0AVG5H00TMfqPjDs0AFG5HDdr7tznjwxPH010AdW5HD4nHbYPjczn17xnH0krNtznjRkg1cLnjbzPj6vrHnLg100TgKGujYs0Z7Wpyfqn0KzuLw9u1Ys0A7B5HKxn0K-ThTqn6KsTjYs0A4vTjYsQW0snj0snj0s0AdYTjYs0AwbUL0qn0KzpWYs0Aw-IWdsmsKhIjYs0ZKC5H00ULnqn0KBI1Ykn0K8IjYs0ZPl5fK9TdqGuAnqTZnVuLGCXZb0pywW5R9rf6KspZw45fKYmgFMugfqPWPxn7tkPHn0IZN15HT3njD3PjRdnjm1nH0dnHfvrjb0ThNkIjYkPWR1rj0LPWRkn1n10ZPGujY3mhcYuHT3P10snj03uy7B0AP1UHdjnbfzrDRdwH0vPYfYfYND0A7W5HD0TA3qn0KkUgfqn0KkUgnqn0KlIjYz0AdWgvuzUvYqn7tsg1Kxn7ts0Aw9UMNBuNqsUA78pyw15HKxn7tsg1Kxn0Ksmgwxuhk9u1Ys0AwWpyfqn0K-IA-b5iYk0A71TAPW5H00IgKGUhPW5H00Tydh5H00uhPdIjYs0A-1mvsqn0K9uAu_myTqnfK_uhnqn0KbmvPb5Hb3rDNArjDzPWu7PDcvPjTLP1mLPWc1nY7DPDwKfHckxj00IZF9uARqPWnsnW0z0AFbpyfqP1mvnYR3wWTLn1fLPHTLPWbvwW0znj-jrHujP1-DwjD0mMfqn0KEmgwL5H00ULfqn0KETMKY5H0WnanWnansc10Wna3snj0snj0Wnansc10WQinsQW0snj0snankQW0snjDsn0K3TLwd5HmdnH01njf0TNqv5H08Pj9xna3sn7tsQW0sg108nHIxna3kPdtkQW0sg108nHIxn0KBTdqsThqbpyfqn0KzUv-hUA7M5H00mLmq0A-1gvPsmHYs0APs5H00ugPY5H00mLFW5HfknjDz&us=newvui&xst=m1Y4rj97wW6knWmvwHwaPWfLP1TvP1mzn1PKwjwDfRDzngss0ycqP1mvnYR3wWTLn1fLPHTLPWbvwW0znj-jrHujP1-DwjDKT1Yknj0vPWbzPHm1Pj03PWR1rjbzndtznWNxn07L5yPWpzY31eBSSo1__8Q2dQ1K8V0KTHdWmv_3zVitsUof0gRqPWRknjnsP07Y5HDvPHn3njTvPHDKUgDqn0cs0BYKmv6quhPxTAnKUZRqn07WUWYvPH03nHfknNt3gvq-UNqbusD1njcLrjTsnjT3&word=&ck=5482.19.1653807669654.0.0.384.179.0&shh=www.baidu.com&sht=65081411_8_oem_dg&wd=" \o "http://www.baidu.com/baidu.php?url=a00000KEJeCxDFezE_NsPdyeYHgNugM0rrfUCXu1DKfhNTFK_maFCeFm3EUk8PMRRlTpMouxFtsMZ3HGEIihexG32Zi_e1rVF7LHcYm3_CBfwDSLnVatKk7kwGjzni9ZrPvztXvN99ZgUTtXsP7NrC2FHIX7Q5qoQstrGC2urBXCFrr1bB1QZV2XhHY3SCrbBnDT7-mA0BzECcaF8Qj-VC3Pj0Y0.7Y_NR2Ar5Od663rj6tV2UCpblRqArunTxF0S2GqeljG_OpS9KahWw1s8swRZ4_OpS9MvTStTYQ7qvXdmhrMIu_oe2lSguz8HG4UVis1f_unhOmC.U1Yk0ZDqmvPornoREnAlY0Kspynqn0KsTv-MUWYvPHN9nvDYnHwBnA7-uhn1PW-9PhP-rAmkmWbkmHDdn6KY5yPWpzRzwjW58QelErzz3_oREnAlY0KGUHYznWR0u1dEugK1n0KdpHdBmy-bIykV0ZKGujYY0APGujYknH00UgfqnH0kPdtknjD4g1csPH7xnW0vn-t1PW0k0AVG5H00TMfqPjDs0AFG5HDdr7tznjwxPH010AdW5HD4nHbYPjczn17xnH0krNtznjRkg1cLnjbzPj6vrHnLg100TgKGujYs0Z7Wpyfqn0KzuLw9u1Ys0A7B5HKxn0K-ThTqn6KsTjYs0A4vTjYsQW0snj0snj0s0AdYTjYs0AwbUL0qn0KzpWYs0Aw-IWdsmsKhIjYs0ZKC5H00ULnqn0KBI1Ykn0K8IjYs0ZPl5fK9TdqGuAnqTZnVuLGCXZb0pywW5R9rf6KspZw45fKYmgFMugfqPWPxn7tkPHn0IZN15HT3njD3PjRdnjm1nH0dnHfvrjb0ThNkIjYkPWR1rj0LPWRkn1n10ZPGujY3mhcYuHT3P10snj03uy7B0AP1UHdjnbfzrDRdwH0vPYfYfYND0A7W5HD0TA3qn0KkUgfqn0KkUgnqn0KlIjYz0AdWgvuzUvYqn7tsg1Kxn7ts0Aw9UMNBuNqsUA78pyw15HKxn7tsg1Kxn0Ksmgwxuhk9u1Ys0AwWpyfqn0K-IA-b5iYk0A71TAPW5H00IgKGUhPW5H00Tydh5H00uhPdIjYs0A-1mvsqn0K9uAu_myTqnfK_uhnqn0KbmvPb5Hb3rDNArjDzPWu7PDcvPjTLP1mLPWc1nY7DPDwKfHckxj00IZF9uARqPWnsnW0z0AFbpyfqP1mvnYR3wWTLn1fLPHTLPWbvwW0znj-jrHujP1-DwjD0mMfqn0KEmgwL5H00ULfqn0KETMKY5H0WnanWnansc10Wna3snj0snj0Wnansc10WQinsQW0snj0snankQW0snjDsn0K3TLwd5HmdnH01njf0TNqv5H08Pj9xna3sn7tsQW0sg108nHIxna3kPdtkQW0sg108nHIxn0KBTdqsThqbpyfqn0KzUv-hUA7M5H00mLmq0A-1gvPsmHYs0APs5H00ugPY5H00mLFW5HfknjDz&us=newvui&xst=m1Y4rj97wW6knWmvwHwaPWfLP1TvP1mzn1PKwjwDfRDzngss0ycqP1mvnYR3wWTLn1fLPHTLPWbvwW0znj-jrHujP1-DwjDKT1Yknj0vPWbzPHm1Pj03PWR1rjbzndtznWNxn07L5yPWpzY31eBSSo1__8Q2dQ1K8V0KTHdWmv_3zVitsUof0gRqPWRknjnsP07Y5HDvPHn3njTvPHDKUgDqn0cs0BYKmv6quhPxTAnKUZRqn07WUWYvPH03nHfknNt3gvq-UNqbusD1njcLrjTsnjT3&word=&ck=5482.19.1653807669654.0.0.384.179.0&shh=www.baidu.com&sht=65081411_8_oem_dg&wd=) | CK04 | Dojindo,China |
|  | G021-1 | Nanjing Jiancheng Bioengineering, China |
| Lactate dehydrogenase assay kit | A020-2 | Nanjing Jiancheng Bioengineering, China |
| Reactive oxygen species Assay Kit | E004-1 | Nanjing Jiancheng Bioengineering, China |
| FITC Annexin V Apoptosis Detection Kit | SH761 | Dojindo, Japan |
| FITC Annexin V Apoptosis Detection Kit I | 556547 | BD Pharmingen™, US |
| PKH26 | MKCN8501 | Sigma-Aldrich, USA |
| One Step TUNEL Apoptosis Assay Kit | C1089 | Beyotime, China |
| 2,3,5-Triphenyltetrazolium chloride | T8877-5G | Sigma-Aldrich, USA |
| Evans blue | [E2129](https://www.sigmaaldrich.cn/CN/zh/product/sigma/e2129) | Sigma-Aldrich, USA |
| Dihydroethidium (DHE) | S0063 | Beyotime, China |

**EMVs extraction and identification**

Harvested HUVECs media samples were transferred into centrifuge tubes and centrifuged at 300g for 30 min at 4°C, then the supernatant was transferred into new centrifuge tubes and centrifuged at 2000g for 30 min at 4°C. Precipitates were discarded and the supernatant was transferred to ultra-high speed centrifuge bottles and centrifuged at 20000g for 70 min at 4°C. After centrifugation, the culture medium was discarded, and the pelleted membrane vesicles were resuspended in 100ul PBS. Suspended EMVs were immediately analyzed or stored at −80°C. The morphology of EMVs was observed by transmission electron microscopy (TEM). According to Brownian motion, the particle size distribution and concentration were evaluated by real-time visual detection using a nanoparticle tracer analyzer (NTA).

The surface of EMVs contains many antigenic epitopes, and CD144 and CD105 are the antibodies used to identify EMVs. The experimental approach was similar to that reported in previous studies [1]. Briefly, extracted MVs were incubated with anti-CD105 for 2 h and then anti-biotin microbeads (Miltenyi Biotec) were added for 15 min. The microbead-labeled MVs were separated from the suspension using a DynaMag-2 magnet (Life technology). MultiSort release reagent (10 μl; Miltenyi Biotec) was added to each sample to lyse the microbeads. The MVs in the liquid were collected as CD105+MVs. CD105+MVs were incubated overnight with antibody CD144 (1:200 dilution; Santa Cruz Biotechnology) and then incubated with Q-dot 655-conjugated rabbit anti-goat IgG for 2 h (1:350 dilution; Life Technologies). Q-dot 655-labeled MVs were considered CD105+CD144+ MVs. All samples were analyzed by the NTA NS300 system (Malvern Instruments).

**Immunofluorescence tracing of PKH26-labeled EMVs**

PKH26 binds a red fluorescent labeling dye with a long lipid tail to the lipid membrane region of extracellular vesicles. This reagent can stain the lipid membrane while maintaining the activity of extracellular vesicles and can be used for tracing EMVs and detecting the uptake of internalized EMVs by receptor cells.

EMVs were co-incubated with PKH26 reagent, and then PKH26-labeled EMVs were obtained by ultra-high-speed centrifugation at 2000g for 70 min at 4°C. AC16 cells were co-cultured with PKH26-labeled EMVs for 24 h, fixed with 4% paraformaldehyde at room temperature for 10 min, stained with DAPI for 5 min, and then visualized by confocal microscopy (Olympus Fluoview FV300) to observe the uptake and internalization of PKH26-EMVs by AC16 cells.

For in vivo experiments, mouse hearts were exposed and 50 μL of PKH26-labeled EMVs (2x10^9/μL) were injected intracardially, and bioluminescence imaging was performed 3 days after injection using the IVIS Animal Live Imaging System (Xenogen, USA).

**CCK-8 assay for cell activity**

AC16 cells were inoculated in 96-well plates (4000 cells/well) with 3 replicate wells per group, and the HR model was established after 24 h of incubation. HR-injured AC16 cells were treated with HR-EMVs, (HR+P)-EMVs and (HR+D)-EMVs for 24 h, followed by changing the culture medium (100ul/well) and adding CCK-8 solution (10ul /well), and incubated for 2 h at 37°C protected from light. The absorbance at 450 nm was measured with an enzyme marker, and the average of the OD values of the 3 wells was taken for calculation (cell survival rate (%) = OD value of treated group/OD value of control group × 100%).

**LDH assay**

Lactate dehydrogenase (LDH) is abundant in the cytoplasm and cannot penetrate the cell membrane under normal conditions, but can be released outside the cell when cells are damaged or apoptotic. The extent of cell damage can be determined by measuring LDH levels in cell supernatants or plasma. HR-injured AC16 cells were co-cultured with HR-EMVs, (HR+D)-EMVs, and (HR+P)-EMVs, respectively, for 24h, and then 20ul of cell supernatant diluted 5 times was taken to act with LDH matrix solution for 15min, and the absorbance at 450nm was measured by enzyme marker, and the average of OD values of 3 wells was taken for calculation. For in vivo experiments, HR-EMVs, (HR+D)-EMVs, (HR+P)-EMVs or PBS were injected intracardially in IR mice, and 20ul of mouse plasma diluted 20-fold was taken after 3 days of reperfusion to detect LDH activity. The formula for calculating LDH in cell supernatant or plasma: LDH (U/L) = (measured OD - control OD) / (standard OD - blank OD) × standard concentration (0.2umol/ml) × 1000 × dilution times).

**DCFH-DA staining for intracellular ROS levels**

When reactive oxygen species (ROS) were present in the cells, the DCFH-DA probe (Nanjing Jiancheng Bioengineering, E004-1) was oxidized to the strong green fluorescent substance DCF, whose fluorescence intensity was proportional to the level of intracellular reactive oxygen species. HR-injured AC16 cells were co-cultured with HR-EMVs, (HR+D)-EMVs and (HR+P)-EMVs respectively for 24 h and then incubated with 10uM DCFH-DA probe for 30 min at 37°C protected from light, followed by washing the cells with PBS to remove residual DCFH-DA, then digested with EDTA-free trypsin and collected from AC16 cells, and finally the ROS levels of AC16 cells were assessed by flow cytometry.

**Flow cytometry detection of apoptosis**

The percentage of apoptotic cells was detected using the Annexin V-FITC/PI kit (Becton, Dickinson and Company, USA). HR-injured AC16 cells were co-cultured with HR-EMVs, (HR+D)-EMVs and (HR+P)-EMVs for 24h, respectively, and the cells were collected. 1x10^5 cells were resuspended with 100ul 1xBinding Buffer, then 5ul Annexin V-FITC and 5ul PI were added and mixed thoroughly, incubated for 15min at room temperature and protected from light, then 400ul 1xBinding Buffer was added to terminate the staining and detected by flow cytometry within 1h. Viable AC16 cells were considered annexin V−/propidium iodide (PI)−, early apoptotic AC16 cells as annexin V+/PI− and late apoptotic AC16 cells as annexin V+/PI+. Cells of annexin V+/PI− or annexin V+/PI+ were added together to calculate the proportion of apoptotic cells.

**Western Blot**

HR-injured AC16 cells were co-cultured with HR-EMVs, (HR+D)-EMVs and (HR+P)-EMVs for 24h, respectively, and then cellular proteins were extracted.

The protein concentrations were measured by using the BCA protein assay kit (Beyotime, China). Equivalent amounts of proteins in samples were separated electrophoretically by 12% SDS-PAGE and transferred onto PVDF membranes. Following blocked for 2h with 5% non-fat dry milk, and then the membranes were incubated with the antibody in a refrigerator at 4 degrees overnight. On the next day, the PVDF membrane was placed on a shaker for rewarming for 30 min at room temperature, then washed with TBS-T for 5 min 3 times. The following incubation with secondary antibodies marked by horseradish peroxidase was performed for 1.5 h in the shaker at room temperature. Finally, after washing with TBS-T for 10 min 3 times, the expression levels of proteins were detected by an enhanced chemiluminescent detective system. The expression of Bax, Bcl2, Cleaved Caspase-3, Tubulin, CCT4, and GAPDH, were in the whole test plan. ImageJ was used for measurement of the intensity of each band and quantified based on the area of the intensity plot.

**Echocardiography**

Mice were randomly divided into 6 groups: sham/IR+PBS, sham/IR+HR-EMVs, sham/IR+(HR+P)-EMVs, 5 mice in each group. Cardiac function was assessed by echocardiography (VisualSonics Vevo 3100) after 3 days of myocardial ischemia-reperfusion. The mice were anesthetized with isoflurane after dehairing. The left ventricular end-diastolic diameter (LVEDD) and left ventricular end-systolic diameter (LVESD) were measured in a two-dimensional long-axis view. The left ventricular ejection fraction (LVEF), left ventricular shortening fraction (LVFS), left ventricular mass, and left ventricular contraction volume were calculated for cardiac function assessment.

**Evans blue-TTC staining**

Mice were randomly divided into 4 groups: sham+PBS, IR+PBS, IR+HR-EMVs, IR+(HR+P)-EMVs, 5 mice in each group. Myocardial ischemia reperfusion 3 days later, myocardial infarct area (IA) and area-at-risk (AAR) were determined by 2,3,5-triphenyltetrazolium chloride (TTC) and Evans blue staining. Specifically, after anesthesia and intubation, the mice was cannulated in the ascending aorta, retrogradely perfused with Evans blue dye (2%) for 1 minute, then washed with PBS to remove excess dye, and the entire heart was frozen at -80°C for 8 minutes and cut into 1 mm slices. Heart sections were soaked in 1.5% TTC solution at 37°C for 15 minutes, followed by soaking in 10% formalin for 2 hours. The infarct zone (Pale color), danger zone (Red color) and non-left ventricular zone (Blue color) of each slice were measured with Image J and averaged for calculation.

**DHE staining for ROS levels in heart tissue**

Mice were randomly divided into 4 groups: sham+PBS, IR+PBS, IR+HR-EMVs, IR+(HR+P)-EMVs, 5 mice in each group. After 3 days of myocardial ischemia-reperfusion, mouse hearts were removed and 8-μm-thick frozen sections were prepared. Heart sections were incubated with 50 μL of diluted dihydroethidium (DHE, Beyotime, S0063) at 37°C for 30 minutes under protection from light according to the instructions, followed by staining with DAPI for 5 minutes. Finally, sections were observed with a fluorescent microscope (Olympus) and ROS levels were measured.

**TUNEL staining assay**

Mice were randomly divided into 4 groups: sham+PBS, IR+PBS, IR+HR-EMVs, IR+(HR+P)-EMVs, 5 mice in each group. After 3 days of myocardial ischemia-reperfusion, the hearts of mice were removed and frozen sections with a thickness of 8 μm were prepared. The assay was performed according to the TUNEL kit operation instructions. The sections were first fixed and embedded in 50 μg/mL proteinase K solution, incubated for 30 min at 37℃ and washed three times with PBS, followed by incubation with TUNEL buffer for 2 h at 45℃ and washed three times with PBS. DAPI containing an anti-fluorescence quencher was added dropwise before blocking. Confocal imaging is taken by time-lapse confocal microscopy (Olympus Fluoview FV300) with a 40X objective at multiple positions along the coverslip, and the apoptosis rate (number of apoptotic cells/total number of cells x 100%) was calculated.

**Lentiviral infection and plasmids transfection**

The lentivirus particles of NC and lncCCT4-2 silencing (LV-sh-lncCCT4-2) plasmids were designed and synthesized by Genechem Company (Shanghai China). lncCCT4-2 overexpression (OE-lncCCT4-2), CCT4 overexpression (OE-CCT4), CCT4 knockdown (sh-CCT4) and their negative control plasmids (OE-NC and sh-NC) were purchased from Genechem Company (Shanghai China). The lentivirus containing the LV-sh-lncCCT4-2 or LV-sh-NC at a functional titer of 7 × 10^8^ TU/ml for multiplicity of infection (MOI) of 20 were used to infect HUVECs to establish stably knockdown cell lines according to the manufacturer’s instructions. 72 h after infection, transduced HUVECs were screened with 2.5 µg/ml puromycin, and the silencing of lncCCT4-2 was analyzed by qPCR to verify the infection efficiency. These HUVECs were subsequently post-treated with propofol to obtain the corresponding EMVs (sh-lncCCT4-2-(HR+P)-EMVs and sh-NC-(HR+P)-EMVs). AC16 cells were transfected with OE-RNA and sh-RNA plasmids by Lipofectamine™ 3000 and P3000™ (Thermo Fisher Scientific, Inc.) in Opti-MEM Reduced Serum Medium (Thermo Fisher Scientific, Inc.). After 48 h of transfection, the overexpression and silencing of target genes were analyzed by qPCR or western blot to verify the transfection efficiency. The shRNA sequences of the lncCCT4-2, CCT4 and negative control are shown in **Table 3**. The full-length sequences of lncCCT4-2 and CCT4 genes are shown in **Supplementary 3 in Additional file 1.**

**Table 3. The sequences of sh-lncCCT4-2 and sh-CCT4 used for transfection**

| **Gene** | **Target Sequence** |
| --- | --- |
| lncCCT4-2:1 | Sh-RNA 1：GCTTATAGTCTGTGGCCTAAG  Sh-RNA 2：GCACCTGTGGTGACAACTACT  Sh-RNA 3：GGTCCTCAACAGACTGCTTCT  Sh-NC: TTCTCCGAACGTGTCACGT |
| CCT4 | Sh-RNA 1：ccCTATGTGTTATTCGTTGTT  Sh-RNA 2：gcTTTCTCCAATGAGTGTAAA  Sh-RNA 3：ccTGAAGTTGTATTGAAACAA  Sh-NC: TTCTCCGAACGTGTCACGT |

**Real-time quantitative polymerase chain reaction (RT-qPCR)**

Total RNA from AC16 cells was extracted using Trizol, according to the manufacturer’s protocol (Invitrogen), while total RNA from EMVs was extracted using the miRNeasy Mini Kit (QIAGEN Sample and Assay Technologies, Germany), according to the manufacturer’s instructions. And then total RNA was transcribed into complementary DNAs (cDNAs) using the PrimeScript RT reagent kit (Takara Bio, Inc., Otsu, Japan). RT-qPCR was performed in a 10 μL reaction system containing forward/reverse primers, cDNA, and SYBR Green MasterMix (Applied Biosystems; Thermo Fisher Scientific, Inc.) with three replicates. GAPDH was used as the internal control for mRNA and lncRNA normalization. Relative expression of these RNAs was calculated using the 2–∆∆CT method. All the primers used in the study were listed in **Table 4**.

**Table 4. Real-Time PCR sequences of primers**

| **Gene** | **Species** | **Primer sequence(5′-3′)** |
| --- | --- | --- |
| lncCCT4-2:1 | Human | Forward: 5′- AGTTTTCACCACCTGCCTACT-3′  Reverse: 5′- AGCTCTTCAAAAGCAGGTCCA -3′ |
| CCT4 | Human | Forward: 5′- TGGCACCACATCAGTAGTCATCATTG -3′  Reverse: 5′- TGGCTGGGTCAATCACTTTCATCAC -3′ |
| lnc-TLR5-1:6 | Human | Forward:5′- CGCAAGAACAGACTCATACAGG-3′  Reverse:5′-CTTTATCCAATCTGCCCTCGAT-3′ |
| lnc-HIST4H4-1:1 | Human | Forward:5′-ACCAAATGCCCATTGACGAGA-3′  Reverse:5′-ACAAAACATTCGCTGTAAACCAC-3′ |
| lnc-KCNE1B-3:2 | Human | Forward:5′-CTTCCTGCGTCCATGTGATCC-3′  Reverse:5′-CGCGCAAATTACCCACTCC-3′ |
| lnc-GRSF1-2:3 | Human | Forward:5′-CCGTCTTCTTTACTCACAACACC-3′  Reverse:5′-TGGGATAAGCCAAAGGTGAGC-3′ |
| GAPDH | Human | forward: 5′-GGAGCGAGATCCCTCCAAAAT-3′  Reverse: 5′-GGCTGTTGTCATACTTCTCATGG-3′ |

**References:**

[1]. Wang, J., et al., Analyses of Endothelial Cells and Endothelial Progenitor Cells Released Microvesicles by Using Microbead and Q-dot Based Nanoparticle Tracking Analysis. Sci Rep, 2016. 6: p. 24679.
